# Novel erythrocyte clumps revealed by an orphan gene *Newtic1* in circulating blood and regenerating limbs of the adult newt

**DOI:** 10.1038/s41598-018-25867-x

**Published:** 2018-05-10

**Authors:** Roman M. Casco-Robles, Akihiko Watanabe, Ko Eto, Kazuhito Takeshima, Shuichi Obata, Tsutomu Kinoshita, Takashi Ariizumi, Kei Nakatani, Tomoaki Nakada, Panagiotis A. Tsonis, Martin M. Casco-Robles, Keisuke Sakurai, Kensuke Yahata, Fumiaki Maruo, Fubito Toyama, Chikafumi Chiba

**Affiliations:** 1 Graduate School of Life and Environmental Sciences, University of Tsukuba, Tennodai 1-1-1, Tsukuba, Ibaraki 305-8572 Japan; 2 Biological Division, Faculty of Science, Yamagata University, Kojirakawa 1-4-12, Yamagata, 990-8560 Japan; 3 Department of Biological Sciences, Graduate School of Science and Technology, Kumamoto University, Kurokami 2-39-1, Chuo-ku, Kumamoto 860-8555 Japan; 4 Radioisotope Research Center, Nagoya University, Furo-cho, Chikusa-ku, Nagoya 464-8602 Japan; 5 Department of Anatomical Sciences, Faculty of Allied Health Sciences, Kitasato University, Kitasato 1-15-1, Minami-ku, Sagamihara, Kanagawa 252-0373 Japan; 6 Department of Life Science, Faculty of Science, Rikkyo University, Nishi-Ikebukuro 3-34-1, Toshima-ku, Tokyo 171-8501 Japan; 7 Department of Agri-Production Sciences, College of Agriculture, Tamagawa University, Tamagawagakuen 6-1-1, Machida, Tokyo 194-8610 Japan; 8 Faculty of Life and Environmental Sciences, University of Tsukuba, Tennodai 1-1-1, Tsukuba, Ibaraki 305-8572 Japan; 9 Department of Comparative and Behavioral Medicine, Faculty of Veterinary Medicine, Nippon Veterinary and Life Science University, Kyonan-cho 1-7-1, Musashino, Tokyo 180-8602 Japan; 10 Department of Biology and Center for Tissue Regeneration and Engineering at Dayton, University of Dayton, Dayton, Ohio 45469-2320 USA; 11 Department of Life and Environmental Sciences, University of Tsukuba, Tennodai 1-1-1, Tsukuba, Ibaraki 305-8572 Japan; 12 Graduate School of Engineering, Utsunomiya University, Yoto 7-1-2, Utsunomiya, Tochigi, 321-8585 Japan

## Abstract

The newt, a group of urodele amphibians, has outstanding ability to repeatedly regenerate various body parts, even in the terrestrial life-stage. In this animal, when the limb is amputated, a cell mass named the blastema appears on the stump and eventually gives rise to a new functional limb. Erythrocytes (red blood cells) in most non-mammalian vertebrates, including the newt, preserve their nucleus throughout their life-span, although physiological roles of such nucleated erythrocytes, other than oxygen delivery, are not known. Here we report novel behavior of erythrocytes in the newt. We identified an orphan gene *Newtic1*, whose transcripts significantly increased in the blastema. Newtic1 was expressed in a subset of erythrocytes that formed a novel clump (EryC). EryC formed a complex with monocytes and was circulating throughout the body. When the limb was amputated, EryCs were newly generated in the stump and accumulated into a distal portion of the growing blastema. Our data suggested that the newt erythrocytes carried multiple secretory molecules including growth factors and matrix metalloproteases, and were capable of delivering these molecules into the blastema as a form of EryCs. This study provides insight into regulations and roles of nucleated erythrocytes, that are independent of oxygen delivery.

## Introduction

The newt belongs to the family *Salamandridae* in urodele amphibians^1^. This animal is the only known tetrapod (four-limbed vertebrate) that can regenerate, as an adult in the terrestrial life-stage, their various body parts through reprogramming/dedifferentiation of terminally differentiated cells^2–7^. It is a long-sought issue whether such exceptional ability of the newt can be explained by genes conserved in vertebrates including humans, or by unique genes that the newt may have evolved^8,9^. In this study, we first investigated unique genes that are specifically expressed in the adult newt limb blastema. We finally screened an orphan gene (we named it *Newtic1*), that was found only in urodele amphibians. Surprisingly, Newtic1 was specifically expressed in a subset of erythrocytes that formed a novel aggregate structure (we named it the erythrocyte clump (EryC)). We investigated behavior of Newtic1-expressing erythrocytes in normal circulating blood and during limb regeneration in both larval and adult newts. This study provides evidence suggesting that Newtic1-expressing erythrocytes contribute toward limb regeneration specifically in the terrestrial life-stage. Furthermore, to our knowledge, this study provides for the first time insight into regulations and roles of nucleated erythrocytes in non-mammalian vertebrates, that are independent of oxygen delivery.

## Results

### Identification of Newtic1

**Figure 1:**
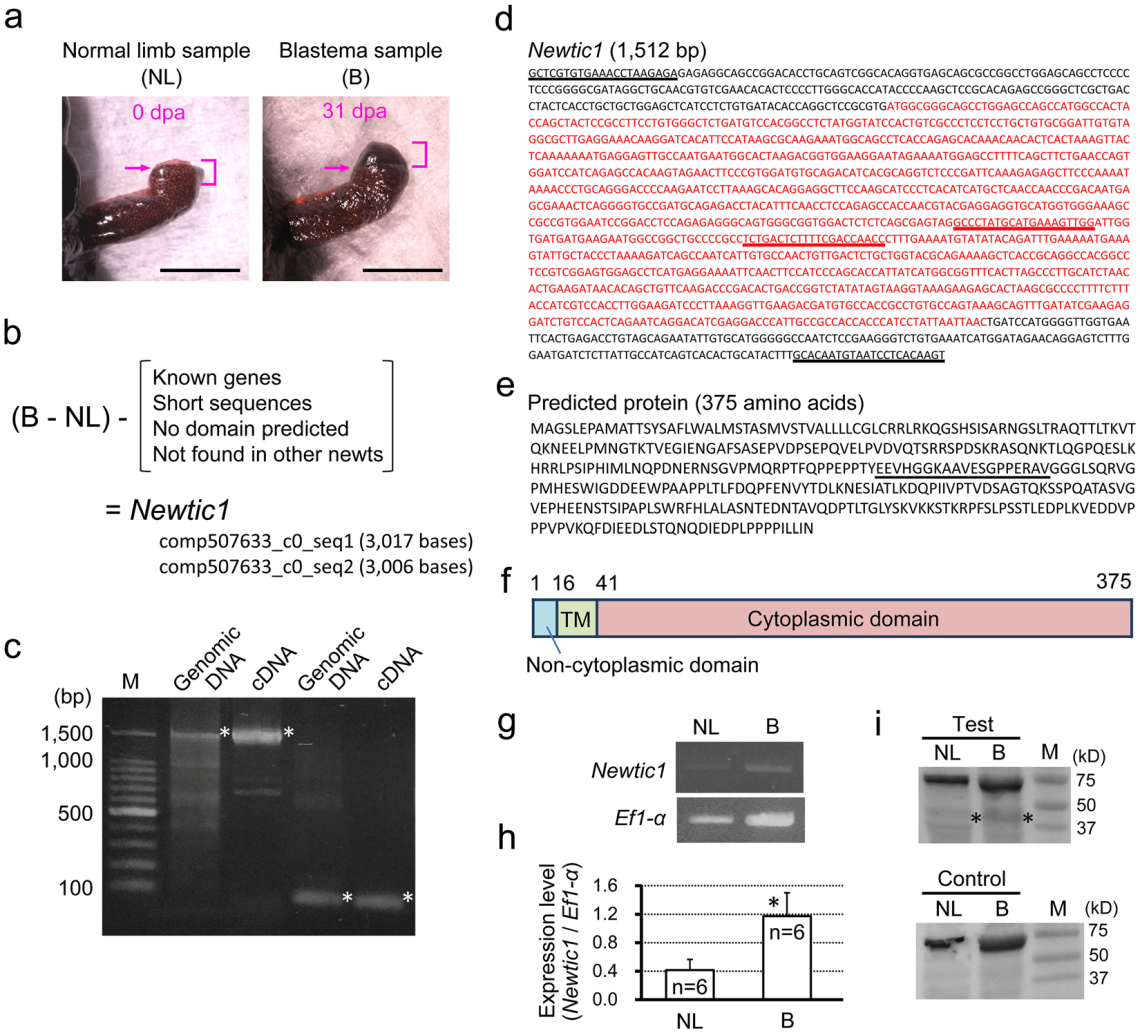
Identification of Newtic1. (**a**) Tissue sampling. For normal limb (NL), tissues in the region up to 1.5 mm proximal to the amputation plane were harvested immediately after the limb was amputated in the middle of the forearm (0 day post-amputation (dpa)). For the blastema (B), samples at stages I to III (see Fig. 4a) were harvested by sectioning the limb at the location of amputation. Scale bars, 5 mm. (**b**) *In silico* screening of unique genes specific to the blastema (Supplementary Figures S3 and S4). We finally obtained a gene *Newtic1*. (**c**) Confirmation of the existence of *Newtic1* (n = 3). PCR products (1,512 bp and 76 bp) were obtained in both genomic DNA and blastema cDNA (asterisks). Bands were separated on the same gel. M: marker. (**d**) The confirmed sequence of *Newtic1*. It was present in both the genome and cDNA. Red: open reading frame. Underlines: sequences corresponding to the primer sets for PCR in (**c**,**e**) Amino acid sequence of a deduced protein. Underline: epitope sequence of Newtic1 antibody. (**f**) Secondary structure of the protein. TM: transmembrane domain. (**g**,**h**) Expression levels of *Newtic1* in NL and B. *Newtic1* was amplified by PCR with NL and B cDNA samples (n = 6 each). *Ef1-α* was also amplified as an internal control. Full-length gel images for the data are shown in Supplementary Figure S6a. The quantity of the PCR product of *Newtic1* was normalized by that of *Ef1-α* in the same cDNA sample. The mean expression level of *Newtic1* in B was significantly higher than that in NL (Student’s *t*-test, *p < 0.05). Of note, weak *Newtic1* expression was detected, as shown in (**g**), in 4 of 6 NL samples. (**i**) Presence of Newtic1 protein. Western blotting was carried out with the same volume of protein samples which were extracted from the same weight of NL and B samples (n = 3). The NL and B protein samples were blotted on the same membrane. Using Newtic1 antibody (Test), weak protein bands corresponding to Newtic1 (40.7 kD) were detected in both samples (asterisks), although the band intensity in the blastema was slightly higher. For the control (Control), Newtic1 antibody was replaced with RFP antibody. The membranes for Test and Control were stained separately. The 60–70 kD bands were caused by a non-specific reaction of the secondary antibody. M: marker.

We first constructed a *de novo* assembled transcriptome database, named TOTAL, which encompassed genes of the Japanese fire-bellied newt *Cynops pyrrhogaster* (see Supplementary Figures S1 and S2). This database is now available on the sequence resource site IMORI (http://antler.is.utsunomiya-u.ac.jp/imori/). Utilizing TOTAL and other databases on IMORI, as well as the original mRNA-seq data sets on NCBI (SRP034152), we investigated genes whose transcripts increased in association with blastema formation, and finally identified *Newtic1* (1,512 bp; GenBank MG923550) which encodes a 40.7 kD transmembrane protein (Fig. 1; see Supplementary Figures S3 and S4, and Supplementary Table S1). We found a *Newtic1* ortholog in another newt species *Notophthalmus viridescens*, but did not identify any homologous genes in other organisms, including in axolotl (*Ambystoma mexicanum*) and frogs. Therefore, until recently, this gene was assumed to be specific to the newt. However, recent publications of the genome and transcriptome data sets in urodeles enabled us to discover *Newtic1* orthologs in axolotl^10^ and in another newt species *Pleurodeles waltl*^11^ (Supplementary Table S2 and Supplementary Figure S5). Consequently, we conclude that *Newtic1* is an orphan gene that might have appeared in a clade of urodele amphibians. In other words, newt-specific protein-coding genes involved in blastema formation are very limited, if present.

### Newtic1 is specifically expressed in a subset of erythrocytes

**Figure 2:**
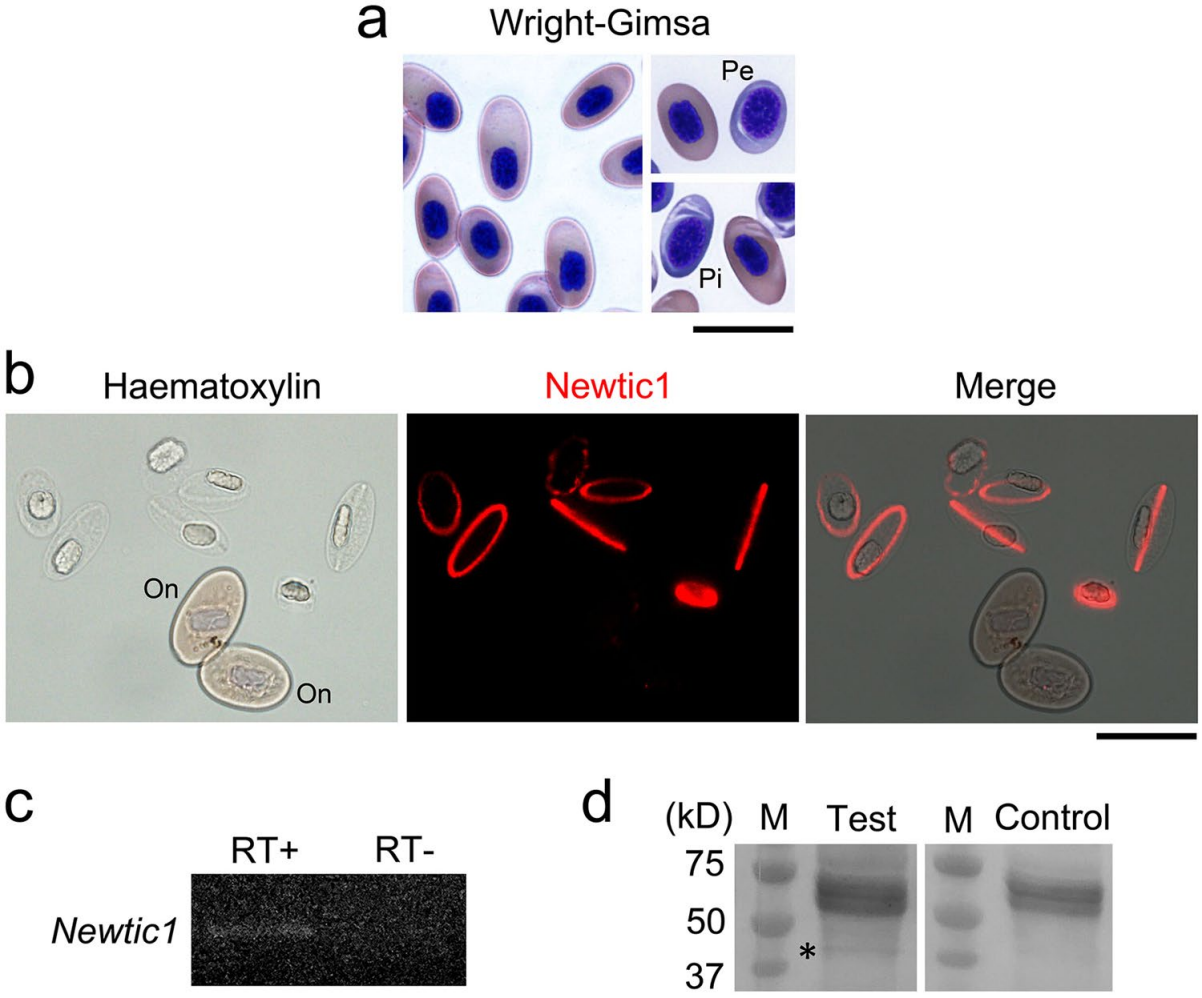
Newtic1 is specifically expressed in a subset of erythrocytes. (**a**) Adult newt erythrocytes. Wright-Giemsa stain of blood smear (peripheral blood) revealed a large number of nucleated erythrocytes (normoblasts) (n = 10; Supplementary Figure S7). Most of them (~99%) were polychromatic normoblasts (PcNobs) at different developmental stages. PcNobs were mostly pink/orange in color of their cytoplasm (late stage), but sometimes grey (intermediate stage; Pi) or blue (early stage; Pe) (for definitions, see Supplementary Figure S7). Scale bar, 40 μm. (**b**) Newtic1 immunoreactivity in blood cell suspensions (n = 9). Under this set of experimental conditions, almost all PcNobs became slightly swollen and transparent. Newtic1 immunoreactivity was exclusively observed along the equator of those PcNobs. Nuclei were lightly stained with haematoxylin. On: presumptive orthochromatic normoblasts (Supplementary Figure S7). Scale bar, 40 μm. (**c**) Confirmation of the presence of *Newtic1* transcripts in blood cells (n = 3). Total RNA was purified from whole blood cells (proportion of normoblasts was estimated 83–94%). The 497 bp sequence of *Newtic1* was amplified by RT-PCR (RT+), but not without reverse transcription (RT−). Products were electrophoresized on the same gel (for full-length image, see Supplementary Figure S6b). (**d**) Confirmation of the presence of Newtic1 protein in blood cells (n = 3). Western blotting was carried out with protein samples purified from whole blood cells. Newtic1 antibody (Test) labeled a band corresponding to Newtic1 protein (40.7 kD; asterisk), while RFP antibody (Control) did not. The membranes for Test and Control were stained separately. The 60–70 kD bands were caused by a non-specific reaction of the secondary antibody. M: marker.

To identify cells expressing Newtic1, we carried out immunohistochemistry in blastema. Unlike our initial expectation that such cells would be dedifferentiated cells, we wondered if these cells might occur in blood. Therefore, the study was reset by characterizing the blood components (Supplementary Figure S7), and finally concluded that Newtic1 is specifically expressed along the equatorial plane at the periphery of premature erythrocytes (polychromatic normoblasts: PcNobs; Fig. 2).

It is known that vertebrates in good health, except for mammals including humans, have nucleated erythrocytes in blood that circulate throughout the body^12,13^. Such nucleated erythrocytes correspond to PcNobs or orthochromatic normoblasts (OcNobs) in humans. In adult newts, normoblasts are produced in the spleen and mature in blood vessels during circulation^12–16^. In peripheral blood, we found various developmental stages of normoblasts (Fig. 2a and Supplementary Figure S7), which accounted for 83–94% in all blood cells (n = 9). The proportion of basophilic normoblasts (immature state) and PcNobs in all normoblasts was less than 2.2% (1.1 ± 0.3%, n = 9) and more than 97.8% (98.9 ± 0.3%, n = 9), respectively. Immunolabeling of blood cells revealed the expression of Newtic1 exclusively in PcNobs, but not all PcNobs were positive (25.4 ± 12.4%, n = 9). Moreover, the proportion of Newtic1-positive (Newtic1+) PcNobs tended to decrease as the PcNobs matured (early stage, 32.0 ± 6.0%; intermediate/late stage, 23.0 ± 4.0%; Student’s *t*-test, p = 0.0321).

### Newtic1-expressing erythrocytes form an aggregate structure EryC, which is accompanied by monocytes and circulating throughout the body

**Figure 3:**
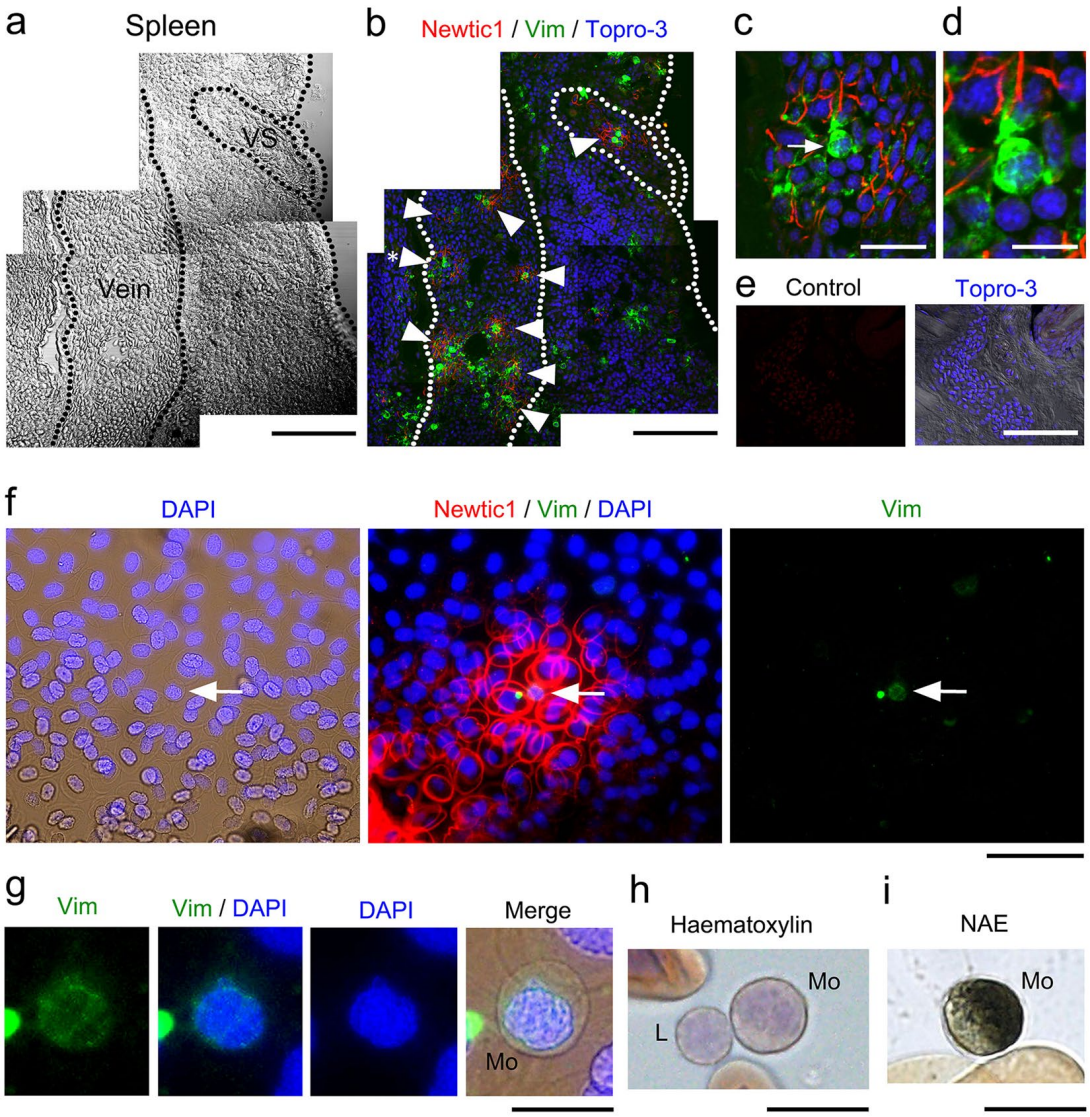
Newtic1-expressing erythrocytes form an aggregate structure EryC, which is accompanied by monocytes and circulating throughout the body. (**a**–**d**) Representative images showing EryCs in the spleen (n = 3). (**a**) A section of the spleen. VS: Venus sinus. Scale bar, 200 μm. (**b**) Immunofluorescence labeling of the same section with Newtic1 antibody (red) and vimentin antibody (green). Arrowheads point EryCs. Topro-3: nucleus. Scale bar, 200 μm. (**c**) Enlargement of EryCs (asterisk in **b**). EryCs were accompanied by spherical cells with vimentin-immunoreactivity (arrow). Scale bar, 40 μm. (**d**) Enlargement of a spherical cell (arrow in **c**). Scale bar, 40 μm. (**e**) Negative control for the immunofluorescence labeling of blood-containing vessels (n = 3). Control: labeling with RFP antibody (red). Topro-3: nucleus. Scale bar, 200 μm. (**f**,**g**) Vimentin-immunoreactive (Vim+) cells in EryCs. (**f**) Immunofluorescence labeling of blood cells with Newtic1 antibody (red) and vimentin antibody (green). Peripheral blood was collected in a plastic dish and allowed to coagulate to preserve the structure of EryCs. Newtic1-immunoreactivity was only observed in PcNobs of EryCs, but not in free PcNobs scattered around EryCs. Arrow: Vim+ cell. DAPI: nucleus. Scale bar, 80 μm. (**g**) Enlargement of the Vim+ cell (arrow in **f**). Vim+ cells were 16–20 μm in diameter and had a brain-shaped nucleus surrounded by vimentin. From these morphological characteristics, Vim+ cells were identified as monocytes (Mo; see below). Note that Vim+ cells in blood were always monocytes. Scale bar, 20 μm. (**h**) Representative image of haematoxylin stain of the adult newt monocyte (Mo) and lymphocyte (L). Scale bar, 20 μm. (**i**) Example image of adult newt monocytes identified by α-naphthyl acetate esterase (NAE) activity in their cytoplasm. Scale bar, 20 μm.

In tissue sections of normal adult newts, we found that the blood stream in thick vessels contained an aggregate structure of Newtic1+ PcNobs, and these were named the erythrocyte clump (EryC) (Fig. 3). EryCs were typically accompanied by one or two spherical cells that exhibited immunoreactivity to vimentin intermediate filaments at high levels (Fig. 3b–d). The vimentin-immunoreactive (Vim+) cells were identified as monocytes (Fig. 3f–i). It is noteworthy that such EryC-monocyte complexes were frequently observed in the veins of the spleen (Fig. 3a,b), suggesting that normoblasts produced in the spleen remain temporarily in veins to form EryC-monocyte complexes, and the complexes circulate thereafter together with other blood components, including free Newtic1-negative PcNobs. The minimum size of an EryC-monocyte complex is comprised of 1–2 monocytes and 6–10 PcNobs.

### EryCs are newly generated in the limb stump and accumulated in a distal portion of the blastema

**Figure 4:**
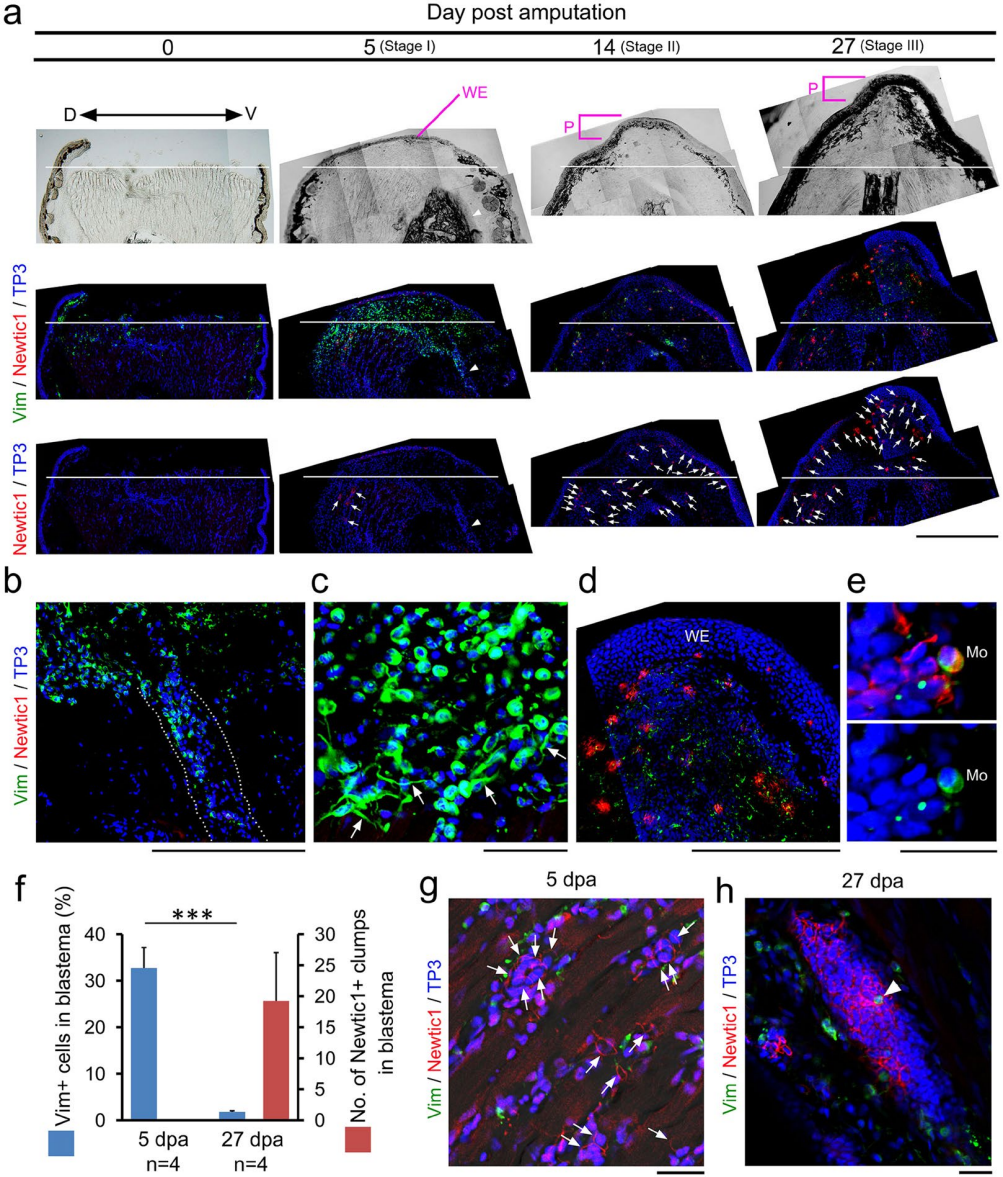
EryCs are newly generated in the limb stump and accumulated in a distal portion of the blastema. (**a**) Change in distribution pattern of EryCs and vimentin-immunoreactive (Vim+) cells during blastema formation (n = 4 each). Arrows indicate EryCs. Arrowheads in 5 dpa indicate a large blood vessel containing Vim+ cells. White line: amputation plane. WE: wound epidermis. P: protrusion. Topro-3 (TP3): nucleus. D: dorsal side. V: ventral side. Scale bar, 1 mm. (**b**) Enlargement of the blood vessel (arrowhead in **a**). Dotted lines indicate the wall of the vessel. Vim+ cells in the vessel, which were exclusively monocytes, seemed to be released from the open-end of the vessel into the blastema. Scale bar, 500 μm. (**c**) Enlargement of Vim+ cells in the blastema at 5 dpa in (**a**). Almost all of the Vim+ cells were morphologically identified as monocytes. Some cells with long processes (arrows) that gathered around the amputation plane also showed vimentin immunoreactivity. Scale bar, 100 μm. (**d**) Enlargement of the protrusion of blastema at 27 dpa in (**a**). Scale bar, 500 μm. (**e**) Representative image showing monocytes (Mo) in the blastema at 27 dpa (n = 3). Scale bar, 50 μm. (**f**) The proportion of Vim+ cells and the number of Newtic1+ clamps in a blastema at 5 and 27 dpa. At 5 dpa, Vim+ cells (178–283/section) occupied ~32.8% of cells in the blastema, while Newtic1 immunoreactivity did not appear in the blastema. In contrast, at 27 dpa, a large number of Newtic1+ clamps were distributed in the blastema (11–32/section), while the proportion of Vim+ cells in the blastema (7–17/section) declined to ~1.8% (Welch’s *t*-test, ***p < 0.001; see Methods). (**g**) Appearance of Newtic1 immunoreactivity in PcNobs accumulated in dilated capillaries in the region proximal to the amputation plane at 5 dpa. We enhanced the red signal in the image at 5 dpa in (**a**) to reveal weak fluorescence of Newtic1 immunoreactivity. Arrows point to Newtic1-immunoreactive PcNobs in dilated capillaries in between muscle fibers. Scale bar, 50 μm. (**h**) Accumulation of Newtic1-immunoreactive PcNobs in thick blood vessels in the region proximal to the amputation plane in the regenerating forearm at 27 dpa (n = 3). Monocytes were occasionally observed in the blood stream (arrowhead). Scale bar, 100 μm.

We analyzed the changes in the distribution pattern of Newtic1 immunoreactivity during adult newt limb regeneration (Fig. 4; Supplementary Figure S8). In intact forearms of the forelimbs, EryC-monocyte complexes were occasionally observed in thick blood vessels such as radial and ulnar arteries and those along the sub-epidermal layer in the skin, but not in thin blood capillaries within the tissues. No other cells showed Newtic1 immunoreactivity. When the forelimb was amputated in the middle of the forearm, bleeding stopped within a few minutes by coagulation. After bleeding stopped, we carefully removed the clot, which was still soft and jelly-like, from the surface of the wound. At this stage, EryCs were hardly observed in the amputated forearm (day 0 in Fig. 4a). Between 3 and 5 days post amputation (dpa), the wound was closed by the epithelium (wound epidermis) which had grown from the wound edge of the skin of the stump. Following wound closure, a large number of monocytes gathered from blood vessels into the space underlying the wound epidermis, contributing to early blastema (stage I; 5 dpa in Fig. 4a–c). Vim+ cells with long processes were localized on the stump ends of tissues (Fig. 4c). These cells are probably macrophages that are differentiating from monocytes. At this stage, we first recognized a small number of EryCs in the dorsal side of the stump region (5 dpa in Fig. 4a). In this study, we defined the stump region as the region 0–1 mm proximal to the amputation plane. It is important to note that these EryCs were not accompanied by monocytes.

When the top of the blastema started to protrude (stage II), the number of Vim+ cells in the blastema decreased dramatically (14 dpa in Fig. 4a,f). Instead, we first recognized EryCs within the blastema while observing an increase in the number of EryCs in the stump region. As the blastema grew, EryCs in it increased in number and size, and accumulated in the protrusion of the blastema (stage III; 27 dpa in Fig. 4a,d,f). On the other hand, the number of EryCs in the stump region decreased. In this stage, we occasionally observed Vim+ monocytes in the blastema (Fig. 4e). Blastema samples at stages I to III were used to screen *Newtic1* (Fig. 1).

Since EryCs from stage I to stage II were not accompanied by monocytes, we attempted to assess their origin. In stage I, we detected Newtic1 immunoreactivity at low levels in a large number of PcNobs that accumulated in dilated blood capillaries in the stump region (Fig. 4g). On the other hand, EryC-monocyte complexes were occasionally observed only in the thick blood vessels near the elbow. In addition, the proportion of Newtic1+ PcNobs in peripheral blood did not change (13–34%, 25.5 ± 4.5%, n = 4). These observations suggest that EryCs are formed on site without monocytes. In other words, EryCs in the growing blastema formed independently of those in circulating EryC-monocyte complexes. In stage III, we observed a large number of Newtic1+ PcNobs that were stuck throughout thick blood vessels in the stump region (Fig. 4h). Monocytes were occasionally recognized in these aggregates. This phenomenon may indicate that the diameter of blood capillaries in the stump region recovered after inflammation ended. This would allow EryCs in the capillaries to be extruded into thick blood vessels where normal blood, which contains EryC-monocyte complexes, circulates.

We addressed how EryCs were translocated from the stump region to the blastema across the amputation plane. A reasonable explanation was transportation through regenerating blood vessels/capillaries. In fact, the spatiotemporal changes in distribution pattern of EryCs seemed to agree with previous descriptions of angiogenesis in the blastema^17^. Therefore, we carefully examined blastema tissues and confirmed that EryCs were localized within the blood vessels/capillaries extending from the stump to the blastema (Supplementary Figure S8). Interestingly, as the protrusion appeared in the blastema, leading ends of regenerating blood vessels/capillaries in the protrusion connected with each other to form loops with large ventricles. At this stage, EryCs that had passed through different vessels/capillaries gathered to form larger aggregates in the ventricles (Supplementary Figure S8a,b). The presence of EryC-monocyte complexes in the ventricles at stage III may be explained by restarted circulation concomitant with the formation of loops among blood vessels/capillaries.

As the digits appeared in the regenerating forearm, aggregates of Newtic1+ PcNobs showed an obvious decrease in size and density (Supplementary Figure S8c). In the region proximal to the amputation plane, the distribution pattern of Newtic1 immunoreactivity recovered, similar to intact limbs. EryCs observed at this stage were always accompanied by monocytes (Supplementary Figure S8d–f). This may reflect that EryCs originating from PcNobs in the stump region had been replaced with normal blood as circulation recovered.

### EryCs contribute to limb blastema specifically in the terrestrial life-stage

**Figure 5:**
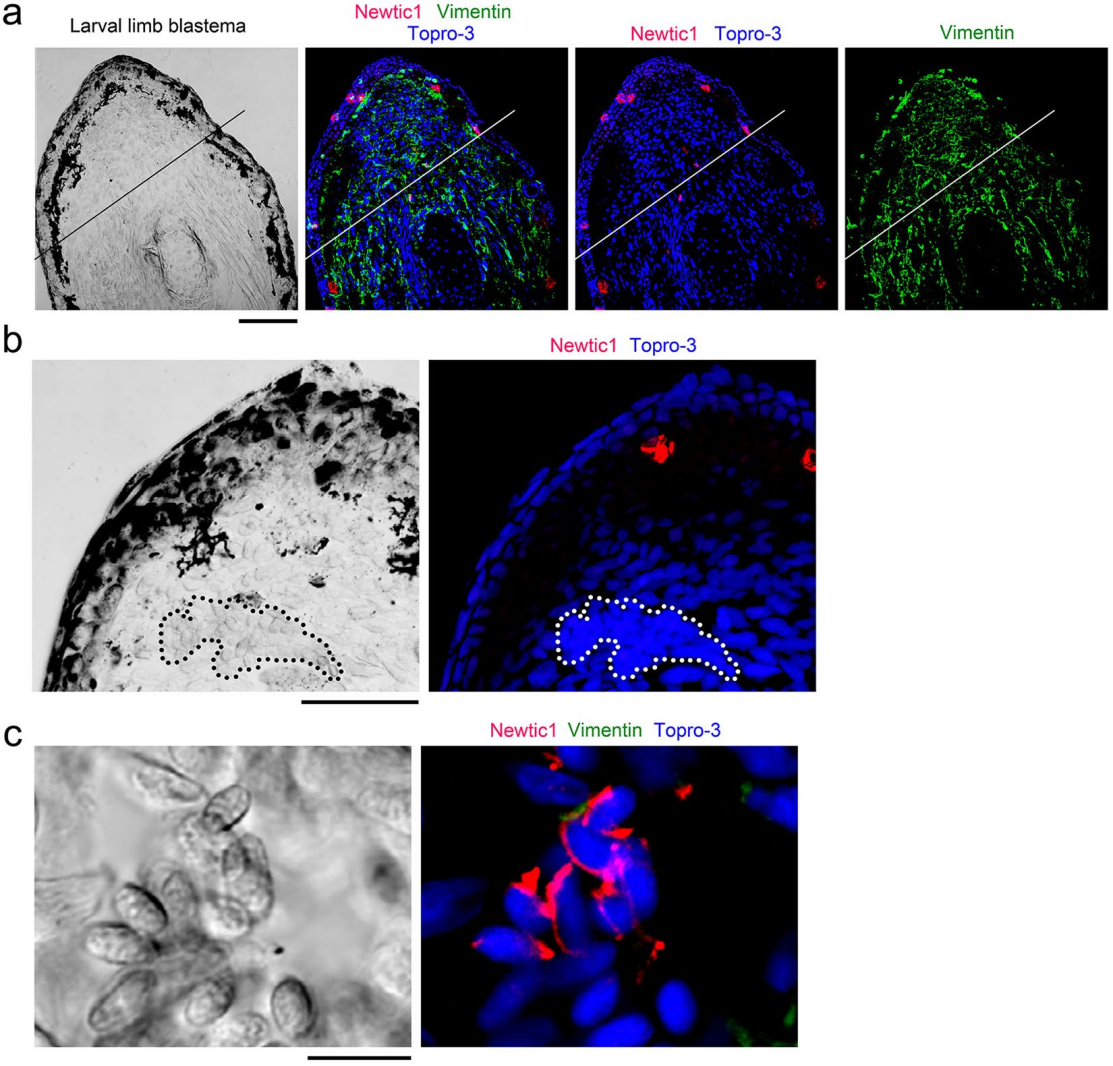
Newtic1-expressing erythrocytes do not contribute to larval limb blastema. (**a**) Representative image showing immunoreactivity in the blastema of the forearm in swimming larvae ~3 months old (stage 57–58; n = 3). Line: amputation plane. In contrast to adult limbs (Fig. 4), Newtic1 immunoreactivity was hardly observed in mesenchymal cells in the blastema, while a large number of fibrous cells were labeled with vimentin antibody. Note that in larval limbs, Newtic1 antibody labeled cells of gland-like structures along the skin (even on the blastema), and vimentin antibody labeled many fibrous cells similar to fibroblasts. Topro-3 (TP3): nucleus. Scale bar, 200 μm. (**b**) A section showing a large blood vessel (dotted line) in the blastema. We were unable to find Newtic1-immunoreactive (Newtic1+) cells in blood capillaries/vessels in the blastema (n = 3). Scale bar, 100 μm. (**c**) Newtic1+ erythrocytes in swimming larvae ~3 months old (stage 57–58). Larval erythrocytes (longer axis: ~15 μm) were about half the size of those from adults. Gathering of Newtic1+ erythrocytes was rarely observed in normal blood (2 of 26 sections from 9 limbs). These cells were not associated with either vimentin-immunoreactive cells or cells such as monocytes. Scale bar, 100 μm.

We addressed whether erythrocytes in larval newts also express Newtic1 because amphibians generally switch primary hematopoietic organs from the kidney to the spleen during metamorphosis^12,13^. In swimming larvae a few weeks before metamorphosis (stage 57–58), we detected Newtic1 immunoreactivity in a small number of erythrocytes in thick blood vessels. These cells rarely gathered and were not accompanied by Vim+ cells (n = 9; Fig. 5). In the limb blastema, large blood vessels containing erythrocytes were observed but Newtic1 immunoreactivity was never detected (n = 3; Fig. 5). These results suggest that larval erythrocytes are capable of expressing Newtic1 and forming aggregates like EryCs, although Newtic1+ erythrocytes do not contribute to the limb blastema. In other words, at least in this species, the contribution of Newtic1+ erythrocytes to the limb blastema is restricted to a post-metamorphic life.

### Erythrocytes carry multiple secretory molecules and are capable of delivering these molecules into the growing blastema as a form of EryCs

**Figure 6:**
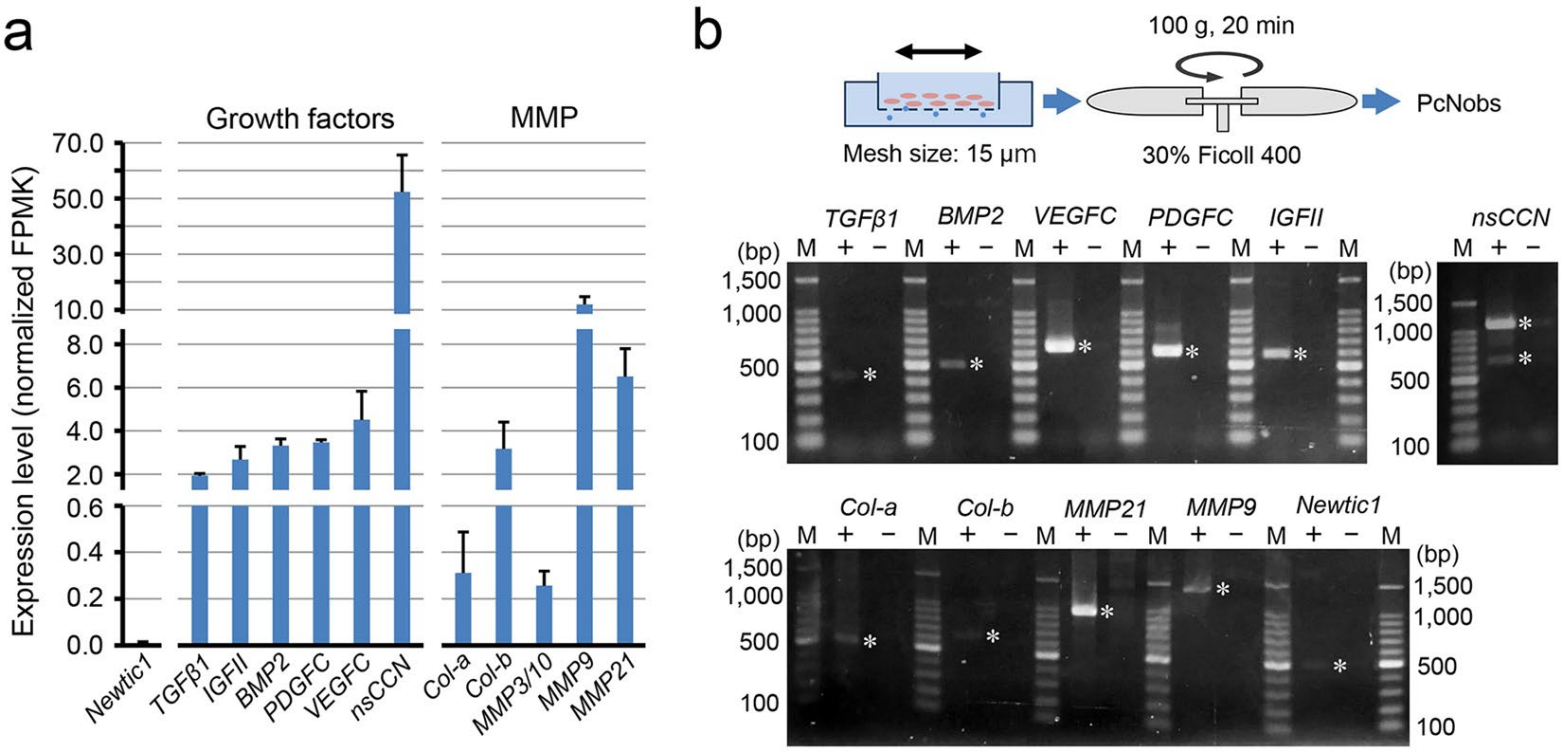
Circulating erythrocytes express genes of a large number of secretory molecules. (**a**) Relative gene expression levels of secretory molecules (growth factors and MMPs) as well as *Newtic1* in whole blood. We carried out mRNA-seq (n = 3) and aligned the sequence reads on TOTAL (Supplementary Table S3). (**b**) PCR confirmation of gene expression in PcNobs (n = 3). PcNobs were purified (99.3–99.8%) from whole blood through a sieve and isopycnic centrifugation (See Methods and Supplementary Figure S9). The representative data set shown here was obtained with the same cDNA sample (+) and its original RNA sample without reverse transcription (−). PCR products (+ and −) of growth factors, those of MMPs and *Newtic1*, and that of *nsCCN* were electrophoresized on the same gel each. We detected the transcription of all of the genes listed in (**a**) (asterisk). As for *nsCCN*, two bands corresponding to the variants were detected. M: marker.

**Figure 7:**
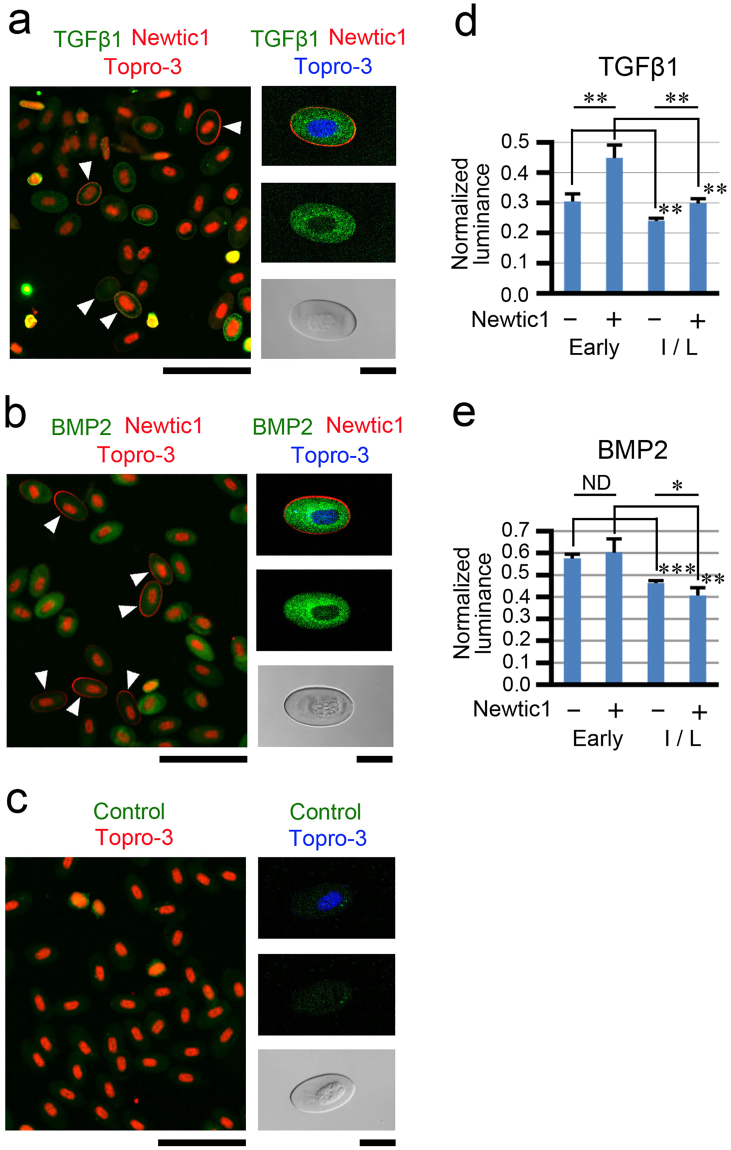
TGFβ1 and BMP2 are expressed in circulating erythrocytes. (**a**) Representative image showing TGFβ1 expression in PcNobs (n = 6). PcNobs in blood cell suspensions were double labeled with TGFβ1 (green) and Newtic1 (red) antibodies. Nuclei were also labeled with Topro-3 (red). Arrowheads point to Newtic1-positive (Newtic1+) PcNobs. PcNobs exhibited TGFβ1 immunoreactivity but their fluorescence intensity was variable. Note that the fluorescence intensity was sometimes higher along the equator of PcNobs, independent of the intensity of Newtic1 immunoreactivity. Scale bar, 100 μm. Right hand column shows a particular Newtic1+ PcNob exhibiting intense TGFβ1 immunoreactivity in its cytoplasm. Upper two panels show confocal images. Scale bar, 20 μm. (**b**) Representative image showing BMP2 expression in PcNobs (n = 3). PcNobs in blood cell suspensions were double labeled with BMP2 (green) and Newtic1 (red) antibodies. The data are presented in the same manner as in (**a**). PcNobs exhibited BMP2 immunoreactivity, but as in TGFβ1, their fluorescence intensity varied. (**c**) Control immunoreactivity (n = 3). PcNobs in blood cell suspensions were labeled with control (RFP; green) antibody. The data are presented in the same manner as in (**a**). (**d**) Relationships of TGFβ1 expression in PcNobs with either their developmental stage or Newtic1 expression. We used representative images obtained from six experiments in (**a**). Total number of PcNobs at an early stage: Newtic1−, n = 51; Newtic1+, n = 41. Total number of PcNobs at an intermediate/late stage: Newtic1−, n = 255; Newtic1+, n = 110. (**e**) Relationships of BMP2 expression in PcNobs with either their developmental stage or Newtic1 expression. We used representative images obtained from three experiments in (**b**). Total number of PcNobs at an early stage: Newtic1-, n = 74; Newtic1+, n = 11. Total number of PcNobs at an intermediate/late stage: Newtic1−, n = 231; Newtic1+, n = 30. Statistical difference: Student’s *t*-test/Welch’s *t*-test; *p < 0.05, **p < 0.01, ***p < 0.001, ND (no difference) (see Methods).

We investigated the potential roles of EryCs in blastema formation of the adult limb. It is known that adult newt PcNobs have granulated cytoplasm containing Golgi apparatus and polysomes as well as mitochondria^15,16^. In addition, our current study on Newtic1 suggests active gene expression in PcNobs. Therefore, we examined the gene expression profile of normal blood by mapping mRNA-seq data (SRP034152; SAMN08574272–08574274) on the TOTAL transcriptome database. The results suggest the expression of a large number of genes encoding secretory molecules (Supplementary Table S3). The list contained growth factors (TGFβ1, IGF-II, BMP2, PDGF-C, VEGF-C, nsCCN) and matrix metalloproteases (Col-a, Col-b, MMP3/10, MMP9, MMP21) (Fig. 6a). We confirmed the transcription of these genes in PcNobs by PCR (Fig. 6b; Supplementary Figure S9), and validated the presence of two growth factors of the TGFβ superfamily (TGFβ1 and BMP2) in PcNobs by immunocytochemistry (Fig. 7). In both growth factors, we found that the level of expression decreased significantly as PcNobs developed (Fig. 7d,e). However, in TGFβ1, expression was higher in Newtic1+ PcNobs throughout development (Fig. 7d), while in BMP2, it was slightly lower in Newtic1+ PcNobs at the intermediate/late stage (Fig. 7e). These results suggest that circulating EryCs, which are composed of Newtic1+ PcNobs at various developmental stages, carry larger doses of TGFβ1 as well as almost average doses of BMP2, relative to the doses carried by the same number of free Newtic1-negative PcNobs. We note that in TGFβ1, the intensity of immunofluorescence was sometimes higher along the equator of PcNobs independent of whether the PcNobs expressed Newtic1 or not.

**Figure 8:**
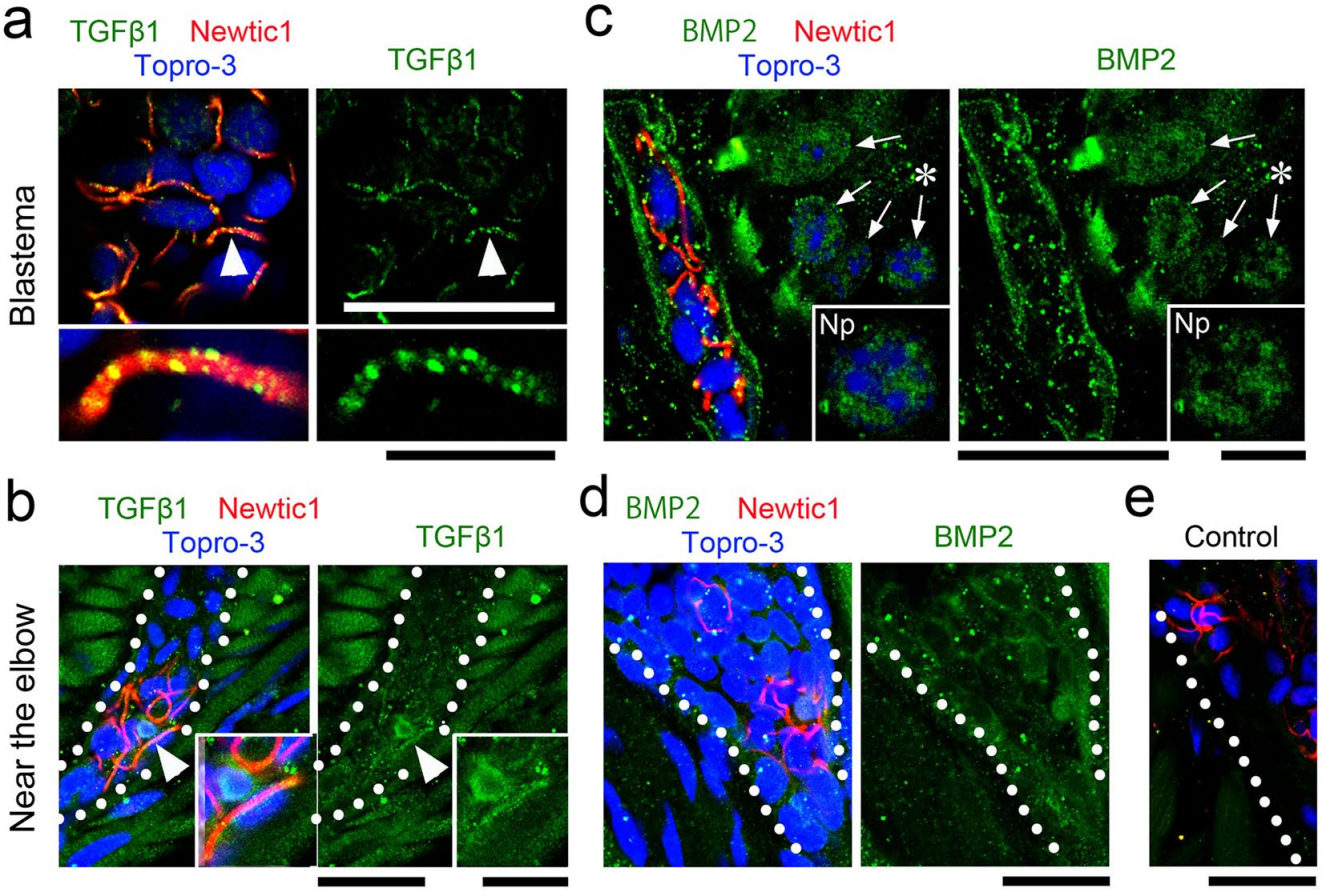
EryCs deliver TGFβ1 and BMP2 into the growing blastema. (**a**) Representative image showing TGFβ1 expression in EryCs in a growing blastema (n = 3). The section was obtained from a regenerating limb in a transitional stage from stage II to stage III. Intense TGFβ1 immunoreactivity was observed in a large number of dots located along the equatorial plane of most PcNobs, though the cytoplasm of PcNobs exhibited weak immunoreactivity. A sample indicated by the arrowhead was enlarged in the lower panel. Topro-3: nucleus. Scale bars, 50 μm (upper) and 10 μm (lower). (**b**) Representative image showing TGFβ1 expression in PcNobs circulating along the thick blood vessels near the elbow (n = 3). The section was obtained from the same limb in (**a**). Dotted lines show the vessel wall. TGFβ1 immunoreactivity in PcNobs was substantially the same as that observed in blood cell suspensions (Fig. 7a), though it was difficult to recognize differences in immunoreactivity between EryCs and free Newtic1-negative PcNobs. Inset: enlarged image of the equatorial plane of PcNob (arrowhead). Scale bars, 50 μm and 10 μm (inset). (**c**) Representative image showing BMP2 expression in EryCs in a growing blastema (n = 3). The section was obtained from a regenerating limb in the transitional stage from stage II to stage III. BMP2 immunoreactivity in the cytoplasm of PcNobs obviously declined while intense immunoreactivity was observed in small dots distributed around the PcNobs as well as along the wall of regenerating capillaries/vessels. Arrows: neutrophil (NP) which exhibited BMP2 immunoreactivity in its cytoplasm. Inset: enlargement of the neutrophil (asterisk). Scale bars, 50 μm and 20 μm (inset). (**d**) Representative image showing BMP2 expression in PcNobs circulating along thick blood vessels near the elbow (n = 3). The section was obtained from the same limb in (**c**). Dotted lines show the wall of the vessel. BMP2 immunoreactivity in PcNobs was substantially the same as that observed in blood cell suspensions (Fig. 7b). Scale bars, 50 μm. (**e**) Example showing control immunoreactivity with Newtic1 (red) and RFP (green) antibodies (n = 3). The image shows tissues near the elbow. Dotted lines show the vessel wall. The section was obtained from the same limb in (**c**,**d**). Scale bars, 50 μm.

We carried out immunohistochemistry of regenerating limbs (Fig. 8; Supplementary Figure S10). We found that immunoreactivities to TGFβ1 and BMP2 in the cytoplasm of PcNobs declined as the PcNobs were translocated into the growing blastema as a form of EryCs, while Newtic1 immunoreactivity in those EryC-forming PcNobs was elevated. However, interestingly, in the case of TGFβ1, we frequently observed intense immunoreactivity in a large number of dots distributed along the equatorial plane of PcNobs (Fig. 8a). In the case of BMP2, immunoreactive dots were also observed in areas surrounding PcNobs, but these were mostly localized along the wall of regenerating capillaries/vessels (Fig. 8c). These changes in the immunoreactivity of EryCs and their surroundings were observed only from stage II. It is generally known that TGFβ superfamily molecules are secreted as a latent factor protected by a large protein cage, which binds to extracellular matrices and elastic microfibrils, and contribute to local concentrations of the latent factor^18^. Taken together, EryCs appear to have released latent TGFβ1 and BMP2 to the internal space of regenerating capillaries/vessels as they entered the region of the growing blastema (Supplementary Figure S11).

## Discussion

In general, circulating erythrocytes are believed to be specialized in the transport of oxygen throughout the body. In adult humans, these cells have lost their nucleus together with other organelles during maturation in the bone marrow, thereby ensuring deformability to pass through even fine blood capillaries in tissues. Nucleated erythrocytes (i.e., normoblasts) are not released in circulating blood in physiologically normal conditions^13^. On the other hand, in non-mammalian species the nucleus of circulating erythrocytes is preserved^12,13^. However, there is little information regarding the physiological roles of such nucleated erythrocytes, except for oxygen transportation. Our current study on *Newtic1* revealed, to our knowledge, for the first time complex behavior of nucleated erythrocytes in normal blood and during limb regeneration. Our results suggest that PcNobs, a dominant nucleated erythrocyte in circulating blood, carry various secretory molecules throughout the body.

In this study, we found that in the newt, PcNobs are capable of expressing Newtic1 throughout all developmental stages, and EryCs are formed by these Newtic1-expressing PcNobs. However, it remains to be determined how Newtic1 expression is selectively induced in a subset of PcNobs. We speculate that monocytes might contribute to this process because EryCs in normal blood are always accompanied by monocytes, and because, during limb regeneration, Newtic1 expression in the stump region begins concomitantly with the accumulation of monocytes in the space between the amputation plane and the wound epidermis. We have yet to determine whether Newtic1 mediates the aggregation of PcNobs. We also do not yet know the evolutionary origin or functions of Newtic1. Newtic1 is predicted to be a transmembrane protein that is localized along the equator of PcNob. However, since this molecule is unlikely to have an extracellular domain, there is the possibility that Newtic1 may associate with a protein complex that mediates cell aggregation, or that Newtic1 expression may be induced by the same signal as that which exists for cell aggregation. In the latter case, Newtic1 might have different functions from cell aggregation.

The physiological roles of Newtic1+ PcNobs or EryCs also remain to be studied. The dose of secretory molecules that EryCs carry, and when and where EryCs deliver those molecules appear to be rigorously controlled. In fact, EryCs carry larger doses of TGFβ1 and average doses of BMP2 in circulating blood, and seem to deliver both factors within the limb blastema. Interestingly, most of the EryC-carrying secretory molecules (TGFβ1, IGF-II, BMP2, PDGF-C, VEGF-C and matrix metalloproteases) predicted in this study are commonly involved in angiogenesis as well as vascular development and remodeling^18–20^. During limb regeneration, EryCs appear in dilated capillaries in the stump region and change their distribution pattern concomitantly with angiogenesis in the growing blastema. Thus, it is reasonable to propose a hypothesis in which EryCs may work toward the regeneration of blood vessels/capillaries. However, importantly, EryCs do not contribute to the larval limb blastema. Therefore, it is necessary to investigate what functions that are specific to limb regeneration in the terrestrial life-stage require EryCs. In addition, the roles of the EryC-monocyte complex that are also specific to the terrestrial life-stage need to be investigated.

Amphibians are generally able to regenerate their limbs in the aquatic life-stage, however their abilities of limb regeneration consistently decline after they have adapted to the terrestrial environment through metamorphosis. Exceptionally, the newt preserves its high regenerative capacity even in its terrestrial life^2,6^. Recently, we demonstrated that the newt switches the cellular mechanism underlying limb muscle regeneration from stem cell-based mechanism to dedifferentiation-based mechanism as it grows beyond metamorphosis^6^. This provided us insight into the presence of mechanisms specific to the terrestrial life-stage. As mentioned above, we demonstrated in the current study that the contribution of EryCs to limb blastema was specific to the terrestrial life-stage, though the presence of newt-specific genes was not evidenced.

In the current study, we listed a lot of candidate secretory molecules that EryCs may deliver (Supplementary Table S3), however we could examine only the two growth factors (TGFβ1 and BMP2) because of availability of antibodies. A recent *in vitro* study suggests a possibility that BMPs digested by serum proteases such as thrombin and plasmin may promote cell cycle re-entry of dedifferentiating skeletal muscle fibers of adult newt limbs^21^. In addition, in adult newt limb regeneration, it has been demonstrated that matrix metalloproteases such as Col-a, MMP3/10 and MMP9 are necessary for blastema formation^22^. As yet, however, it remains to be determined what types of cells provide these secretory molecules. nsCCN, which is a new member of the CCN family growth factor found in the newt, has been assumed to be involved in adult newt heart regeneration^23^. In this case, endocardial cells in the damaged region express nsCCN. Interestingly, our current results suggest that the expression levels of nsCCN in PcNobs were about ten times higher than those of other cytokines (Fig. 6a). PcNobs or EryCs would be the most likely candidates to provide these molecules to an injured region or to the emerging blastema of a limb. It is important to note that molecules released from EryCs could influence not only blood capillaries/vessels but also mesenchymal cells in the blastema because the permeability of growing blood capillaries/vessels can be regulated^24^. In fact, we observed neutrophils among mesenchymal cells in the blastema (Fig. 8c). In addition, it is not surprised that when the limb is amputated, tissues on the wound should be exposed to the contents of PcNobs, because PcNobs are released from the wound and are damaged/punctured during coagulation. In the future, functional studies of PcNobs, Newtic1 and EryCs, as well as the candidate secretory molecules, are necessary to further understand the mechanism that enables the newt to efficiently regenerate the limbs in the terrestrial life-stage.

## Methods

All methods were carried out in accordance with Regulations on the Handling of Animal Experiments in University of Tsukuba (RHAEUT). All experimental protocols were approved by Committee for Animal Experiments in University of Tsukuba (CAEUT). Molecular cloning procedures were approved by the University of Tsukuba Safety Committee for Recombinant DNA Experiments (UTSCRDE; 120119, 170110).

### Animals

The Japanese fire bellied newt *C*. *pyrrhogaster* was used in this study. Embryonic, larval and adult (total body length: male, ~9 cm; female, 11–12 cm) Toride-Imori^25^ were used for the construction of transcriptome databases, molecular cloning, and examination of larval blood and limbs. Adult newts that were captured from Okayama, Kyoto, Fukushima and Miyagi Prefecture by a supplier (Aqua Grace, Yokohama, Japan) were also used for the examination of adult blood and limbs. Animals were reared at 18 °C under natural light conditions^25^. Developmental stages were determined according to established criteria^25^.

### Anesthesia

FA100 (4-allyl-2-methoxyphenol; LF28C054; DS Pharma Animal Health, Osaka, Japan) dissolved in water was used at room temperature (RT: 22 °C). Before surgery, larval (St. 57–58; ~3 months old) and adult newts were anesthetized in 0.05% FA100 for 15 min and 0.1% FA100 for 1–2 h, respectively^6^.

### Surgical operations

After anesthesia, animals were rinsed in distilled water and lightly dried on paper towels. To construct transcriptome databases or for histological analysis, animals were sacrificed to obtain tissue samples^26^; to collect peripheral blood or for the study of limb regeneration, amputation was carried out in the middle of the forearm (at mid-zeugopod region of the forelimb)^6^. Limb amputation involved amputation of one side of the forelimbs of each animal under a dissecting microscope (M165 FC; Leica Microsystems, Wetzlar, Germany) by a blade (for larvae, a tip of the blade (Cat#: 4991482; Feather Safety Razor, Osaka, Japan); for adults, a surgical blade (No. 14; Futaba, Tokyo, Japan)). Larval and adult amputees were allowed to recover in water and moist containers respectively, and then reared in the same conditions. When the regenerating limb reached a desired morphological stage, we carried out the second amputation at the region of the first amputation or in the middle of the upper arm (at the mid-stylopod region) to obtain regenerates for molecular or histological analysis.

### Construction of *de novo* assembled transcriptome databases

For this study, a total of 22 databases were constructed from 19 different tissues, and then integrated into one comprehensive database named TOTAL (Supplementary Figures S1 and S2). For each sample, total RNA was purified and qualified (RIN >8.0) to construct a cDNA library^26^. The cDNA fragments were sequenced (101 bp read x 2) by Illumina HiSeq. 2000/2500 (Hokkaido System Science Inc., Hokkaido, Japan; Sequencing team of KAKENHI 221S0002 in Tokyo University, Kashiwa, Japan)^26^. Cleaned sequence reads were assembled into contigs (*IS*-transcripts) by Trinity (version 2013-11-10; https://github.com/trinityrnaseq/trinityrnaseq/wiki; Trinity.pl–seqType fq–JM 196 G–left $read_file_1.fastq–right $read_file_2.fastq–CPU 30–min_kmer_cov 2–output $output_dirctory_name)^26^. All information is open to the public through IMORI (http://antler.is.utsunomiya-u.ac.jp/imori/).

### *In silico* screening of Newtic1

Newtic1 *IS*-transcripts were obtained through the work flow described in Supplementary Figure S3.

### PCR and molecular cloning

Total RNA was purified from limb tissues or blood samples using Nucleospin kit II (740955.50; Macherey-Nagel GmbH & Co. KG, Düren, Germany), and cDNA was synthesized using Superscript II reverse transcriptase (18064-014; Invitrogen in Thermo Fisher Scientific, Tokyo, Japan) with oligo(dt) 12–18 primers (18418012, Invitrogen in Thermo Fisher Scientific). In the case of limb tissues (Fig. 1g), the mass of blastema samples was weighed and an equivalent mass of intact limb samples was harvested (see Fig. 1a), and these samples were processed for RNA purification and cDNA synthesis at the same scale. Genomic DNA was purified from blood samples using the Wizard Genomic DNA Purification Kit (A1120; Promega, Madison, WI, USA). Using these DNA samples, PCR was carried out with the KODFX system (KFX-101, Toyobo, Osaka, Japan) on an MJ Mini Gradient Thermal Cycler (PTC-1148; Bio-Rad, Hercules, CA, USA). Primer sets used in this study and cycle numbers for the data in figures are listed in Supplementary Table S4. PCR products were subcloned into *Escherichia coli* using a TA cloning system (45-0640, TOPO TA Cloning Kit, Dual Promoter; Thermo Fisher Scientific) and then sequenced by Sanger protocols (ABI 3130; Applied Biosystems, in Thermo Fisher Scientific)^26^.

### Preparation of tissue sections and blood cells

For immunolabeling, larval and adult tissues were fixed in modified Zamboni’s fixative (2% paraformaldehyde (PFA)/0.2% picric acid in phosphate-buffered saline (PBS; pH 7.5)) at 4 °C for 6 h^27^. In some experiments (Supplementary Figure S10), tissues were fixed in 4% PFA in PBS (pH 7.5) at RT for 2 h. Fixed tissues were washed thoroughly with PBS at 4 °C (15 min × 3, 30 min × 3, 1 h, and 6 h), and then allowed to equilibrate in 30% sucrose in PBS at 4 °C. The tissues were embedded in Tissue-Tek® O.C.T. Compound (4583; Sakura® Finetek USA, Inc., Torrance, CA, USA), frozen at about −30 °C in a cryotome (CM1860; Leica), sectioned at about 20 μm thickness, attached on gelatin-coated cover slips, and then dried at RT for a few hours. The sections were stored at −20 °C until use.

Peripheral blood was collected from forelimbs immediately after amputation. For the Wright-Giemsa stain (Fig. 2a and Supplementary Figure S7) and the α-naphthyl acetate esterase stain (Fig. 3i), blood was spread on a glass slide to prepare the blood smear and air dried. For the former stain, the blood on the slide was fixed in approx. 100% methanol for 30 min and air dried. For the latter stain, the blood on the slide was fixed in citrate-acetone-formaldehyde solution (SLBQ6227V; Sigma-Aldrich in Merck, Tokyo, Japan) for 30 sec at RT, and then rinsed thoroughly in running deionized water (DW) for 45–60 sec. These samples were immediately processed for staining (see below). For immunolabeling (Fig. 7a–c), blood was added directly to 4% PFA fixative in a glass bottom dish (D112310; Mastunami, Tokyo, Japan) and then incubated at RT for 2 h. In some experiments (Fig. 2b), blood was fixed in modified Zamboni’s solution at RT for 2 h. Fixed blood cells in suspension were carefully washed with PBS at 4 °C (20 min × 3) and immediately used for labeling. Blood cell suspensions were gently stirred periodically during incubation to prevent coagulation, and solutions were carefully exchanged by micropipettes under a dissecting microscope so as not to lose or select cells. In experiments to examine EryC-monocyte complexes (Fig. 3f,g), blood was put on the bottom of the dish and allowed to coagulate for a few minutes, and then fixed in modified Zamboni’s solution at 4 °C for 6 h. The coagulated blood was carefully washed with PBS at 4 °C (20 min × 3) and immediately used for labeling.

### Blood stain

To characterize blood cells in the adult newt, each blood smear was stained using a standard Wright-Giemsa system (15021; Muto Pure Chemical Co., Ltd, Tokyo, Japan) according to the manufacturer’s instructions. Cell types were identified according to established criteria (Supplementary Figure S6)^12,13,16^. Monocytes were further characterized using an α-naphthyl acetate esterase staining system (Fig. 3i; SLBQ6227V; Sigma-Aldrich in Merck)^28^ according to the manufacturer’s instructions. Immunolabeling of blood cell suspensions was carried out as for tissue sections (see below).

### Antibodies

Antibodies used for immunolabeling in this study are listed in Supplementary Table S5.

### Immunolabeling of tissue sections and blood cells

The same procedures for single or double immunofluorescence labeling were applied to both tissue sections and blood cell suspensions^27^. For single labeling with a certain primary antibody or double labeling with two primary antibodies produced in different species, the following protocol was applied: samples were washed thoroughly (PBS, 0.2% TritonX-100 in PBS, PBS; 15 min each), incubated in blocking solution (5% bovine serum albumin (BSA, 050 M 1599; Sigma-Aldrich in Merck)/2% normal goat serum (S-1000; Vector Laboratories, Burlingame, CA, USA)/0.2% TritonX-100 in PBS) for 2 h, washed as before, and then incubated in primary antibody (antibodies) diluted with blocking solution at 4 °C for 15 h; after washing thoroughly, the samples were incubated in secondary antibody (antibodies) diluted with blocking solution for 4 h and washed thoroughly.

For double labeling with two primary antibodies produced in the same species, entire labeling procedures mentioned above were repeated serially. In the case of double labeling of TGFβ1 and Newtic1, or BMP2 and Newtic1 (Figs 7 and 8), samples were labeled with primary antibody to TGFβ or BMP2 and alexa-488-conjugated secondary antibody. Subsequently, the same samples were labeled with Newtic1 primary antibody and rhodamine-conjugated secondary antibody.

In the case of blood cell suspensions, samples at the bottom of the dish were gently stirred, and solutions were carefully exchanged as for sample preparation. In any labeling protocol, after samples were washed, the nuclei of cells were counterstained with Mayer’s haematoxylin solution (131-09665; Wako Pure Chemical Industries, Ltd., Osaka, Japan), DAPI (1:50,000, D1306; Thermo Fisher Scientific) or TO-PRO-3 Iodide (1:50,000, T3605; Thermo Fisher Scientific). Tissue sections were mounted on a glass slide with 90% glycerol in PBS or into VECTASHIELD mounting medium (H-1000; Vector Laboratories). Tissue sections and blood cell suspensions were immediately subjected to microscopic analysis.

### Western blotting

Protein samples were prepared from blood cells or limb tissues. Cell/tissue samples were collected in lysis solution (25 mM Tris, 150 mM NaCl, 1 mM EDTA-2Na, 1% Igepal CA-630, 1% sodium deoxycholate, 0.1% SDS) containing 1% proteinase inhibitor cocktail (P8340, Sigma-Aldrich in Merck), and protein was extracted according to established protocols^27,29^. In the case of limb tissues (Fig. 1i), in order to control the mass of tissues, we harvested the same weight of blastema samples and intact limb samples (Fig. 1a) in the same volume of lysis solution. After heat-denaturing in 2× sample buffer, immunoblotting was carried out according to established protocols with some modifications^27,29^. In brief, proteins (20 μl each) were separated on 10% gel (456–1033, Mini-PROTEAN TGX Precast Gel; Bio-Rad) by SDS-PAGE, and transferred to an Immun-Blot^®^ PVDF membrane (1620–174; Bio-Rad). Membranes were washed in TBST (100 mM Tris-HCl (pH 7.4), 150 mM NaCl, 0.05% Tween20) for 10 min, incubated in blocking solution (3% BSA in TBST) containing 2% avidin D (Avidin/Biotin Blocking kit, SP-2001; Vector Laboratories) for 1 h, washed in TBST (1 min × 1, 10 min × 1, 20 min × 1), and then incubated with primary antibody diluted in blocking solution containing 2% biotin (Avidin/Biotin Blocking kit) at 4 °C for 15 h. After washing in TBST (1 min × 1, 15 min × 3), membranes were incubated with biotinylated secondary antibody diluted in blocking solution for 90 min at RT. Membranes were washed in TBST, incubated in AB complex (Vectastain ABC Elite kit, PK-6100; Vector Laboratories) in TBST for 90 min, and then washed in TBST. The immunoreaction was visualized using a DAB substrate kit (SK-4100; Vector Laboratories), and then the membranes were washed in chilled distilled water (5 min × 6).

### Gene expression profiling of blood cells

Total RNA was purified from whole blood samples (n = 3) and mRNA-seq was carried out in a manner similar to the construction of transcriptome databases (see above). To investigate the relative expression levels of genes, sequence reads (deposited to NCBI, SRP034152, SAMN08574272-08574274) were aligned along *IS*-transcript sequences in TOTAL using the trinity script align_and_estimate_abundance.pl (–est_method RSEM;–aln_method bowtie2). Subsequently, from 168,047 mapped *IS*-transcripts, we manually screened 304 *IS*-transcripts encoding secretory molecules including growth factors and MMPs (Supplementary Table S3), and then calculated the mean ± SE of expression levels (normalized FPMK) for each secretory molecule gene (Fig. 6a).

Next, to confirm the expression of these genes in PcNobs by PCR (Fig. 6b), we purified PcNobs (>99%) from whole blood cells (Supplementary Figure S9). Whole blood cells collected in PBS were loaded, at 130–200 μl each, onto pluristrainers (15 μm mesh, 43-50015-01; Pluriselect, Germany) placed in 100 ml beakers filled with 40 ml PBS. The strainers containing blood cells were gently shaken in PBS for 15 min and then blood cells in the strainers were mixed using 500 μl micropipettes. This procedure was repeated three times. Subsequently, 3 ml of blood cell suspensions was carefully transferred onto 3 ml of ficoll solution (30% Ficoll PM400 (Type 400; Sigma-Aldrich in Merck) in PBS) in 15 ml tubes. The tubes were then centrifuged at 100 g for 20 min (LC-120; Tomy Seiko Co., Ltd., Tokyo, Japan). PcNobs deposited on the bottom (about 120 μl) were carefully transferred into a microtube. A small quantity (5 μl) of cell suspension was sampled and recovered in PBS to observe its purity under a microscope (Supplementary Figure S9). The remaining pellet of PcNobs was used for cDNA synthesis followed by PCR analysis (see above).

### Image acquisition and analysis

Bands on agarose gels in PCR and protein bands on membranes in western blotting were scanned and quantified by NIH ImageJ software (https://imagej.nih.gov/ij/). Transmitted light and fluorescence images of tissue sections and blood cells were acquired either by a charge-coupled device (CCD) camera system (DP73; cellSens Standard 1.6; Olympus, Tokyo, Japan) attached to a fluorescence microscope (BX50; Olympus) or through a confocal microscope system (LSM510; LSM 5.0 Image Browser software; Carl Zeiss, Jena, Germany). Microscopic images were analyzed by Photoshop CS5 Extended (Adobe Systems, San Jose, CA, USA) as well as with software for the image acquisition systems. In experiments to quantify the concentrations of growth factors in PcNobs (Fig. 7d,e), we selected representative images (for TGFβ1, six images (total number of PcNobs: 40, 53, 71, 84, 89, 120) from six experiments; for BMP2, three images (total number of PcNobs: 88, 123, 135) from three experiments), that were acquired with a 20× object lens by the BX50-DP73 system (for the field of view, see Supplementary Figure S9). For each image, we measured the amplitude of the mean luminance of the cytoplasm of each PcNob against the background using a function in Photoshop, and normalized the values against the maximum in the same image so that data among experiments could be compared. Figures were prepared using Photoshop. Image, brightness, contrast, and sharpness were adjusted according to the journal’s guidelines.

### Cell counting

In blood smear and cell suspensions, the proportion of cells was estimated by counting the number of cells in representative microscopic images (total cell number/view field >50) obtained from at least three independent experiments. The images were acquired with a 20× object lens by the BX50-DP73 system (see above). In tissue sections, representative images were also obtained from at least three independent experiments. Images covering the area to be analyzed were acquired at high resolution through the confocal microscope system, and merged into one image. In experiments to estimate the proportion of Vim+ cells and the number of Newtic1+ clumps in blastema at 5 and 27 dpa (Fig. 4), we examined representative tissue sections from four individuals at each dpa. For these tissues, we sectioned limb samples along the dorso-ventral axis and used the sections around the central axis of the limb. Only cells that showed a clear nucleus were counted. For Vim+ cells, vimentin-immunoreactive cells with a spherical shape, like monocytes (Fig. 3d,g), were counted. For Newtic1+ clumps (i.e., EryCs), clumps composed of at least six Newtic1+ PcNobs were counted.

### Statistics

Data in the text are presented as the mean ± SE. The statistical mean difference was evaluated by a Student’s *t*-test, except when data were not homoscedastic, in which case Welch’s *t*-test was applied, by using Ekuseru-Toukei 2008 software (Social Survey Research Information, Tokyo, Japan).

### Data availability

All relevant cleaned mRNA-seq data have been deposited in NCBI (Sequence Read Archive ID, SRP034152; BioProject ID, PRJNA231688; BioSample ID, SRS515156, SRS589430, SRS685575-685579, SRS685581, SRS685583, SRS685585-685595, SAMN08574272-08574274). The lists of contigs that have been annotated are available in IMORI (http://antler.is.utsunomiya-u.ac.jp/imori/).

## Electronic supplementary material

Supplementary information

Supplementary Table S3
