# Supplementary material for: Novel erythrocyte clumps revealed by an orphan gene Newtic1 in circulating blood and regenerating limbs of the adult newt
Source: Sci Rep. 2018 May 10;8:7455. doi: 10.1038/s41598-018-25867-x (PMC5945717; doi:10.1038/s41598-018-25867-x)
Supplement: Supplementary file 1 — Supplementary information [file 41598_2018_25867_MOESM1_ESM.pdf]

Novel erythrocyte clumps revealed by an orphan gene *Newtic1* in circulating blood and regenerating limbs of the adult newt

Roman M. Casco-Robles, Akihiko Watanabe, Ko Eto, Kazuhito Takeshima, Shuichi Obata, Tsutomu Kinoshita, Takashi Ariizumi, Kei Nakatani, Tomoaki Nakada, Panagiotis A. Tsonis, Martin M. Casco-Robles, Keisuke Sakurai, Kensuke Yahata, Fumiaki Maruo, Fubito Toyama, Chikafumi Chiba

Supplementary Table S1. *IS*-transcripts in the transcriptome of limb blastema (Stage I-III), which correspond to the genes that have been suggested to be involved in limb regeneration

IS-transcript	Gene	Blast description	References
**comp197402_c0_seq1**	RARb	Retinoic acid receptor beta isoform x4	30-33
**comp44533_c0_seq3**	LHX2	LIM/homeobox protein Lhx2 isoform X2	34, 35
**comp27330_c0_seq1**	WNT9A	Wingless-type MMTV integration site family member 9a	36-38
**comp44198_c0_seq2**	SALL1	Sal-like protein 1 isoform x1	39-41
**comp34287_c0_seq2**	ZFHX4	Zinc finger homeobox 4	42
**comp46610_c1_seq1**	MSX1	Msx1 protein	43-45
**comp37101_c0_seq1**	TWT2	Twist-related protein 2	46
**comp25086_c0_seq1**	CRABP1	Cellular retinoic acid-binding protein 1	42, 47-49
**comp122669_c0_seq1**	SHH	Sonic Hedgehog	50-52
**comp46084_c0_seq1**	SCXB	Basic helix-loop-helix transcription factor scleraxis	53
**comp25123_c0_seq1**	FGF10	Fibroblast growth factor 10	54-57
**comp3297_c0_seq1**	FRZ7	Frizzled-7	58

References

30) Giguere, V., Ong, S., Evans, R.M. & Tabin, C.J. Spatial and temporal expression of the retinoic acid receptor in the regenerating amphibian limb. *Nature* **337,** 566–569 (1989).

31) Ragsdale Jr. C.W. *et al.* Identification of a novel Retinoic acid receptor in regenerative tissue of the newt. *Nature* **341,** 654-657 (1989).

32) Carter, C., Clark, A., Spencer, G. & Carlone, R. Cloning and expression of a retinoic acid receptor β2 subtype from the adult newt: evidence for an early role in tail and caudal spinal cord regeneration. *Dev. Dyn.* **240,** 2613-2625 (2011).

33) Nguyen, M. *et al.* Retinoic acid receptor regulation of epimorphic and homeostatic regeneration in the axolotl. *Development* **144,** 601-611 (2017).

34) Showalter, A.D., Yaden, B.C., Chernoff, E.A. & Rhodes, S.J. Cloning and analysis of axolotl ISL2 and LHX2 LIM-homeodomain transcription factors. *Genesis* **38,** 110-121 (2004).

35) Shimokawa, T. *et al.* Lmx-1b and Wnt-7a expression in axolotl limb during development and regeneration. *Okajimas Folia Anat. Jpn.* **89,** 119–124 (2013).

36) Kawakami, Y. *et al.* Wnt/b-Catenin signaling regulates vertebrate limb regeneration. *Genes Dev.* **20,** 3232-3237 (2006).

37) Später D. *et al.* Wnt9a signaling is required for joint integrity and regulation of Ihh during chondrogenesis. *Development* **133,** 3039–3049 (2006).

38) Yokoyama, H. *et al.* Wnt/β-catenin signaling has an essential role in the initiation of limb regeneration. *Dev. Biol.* **306,** 170-178 (2007).

39) Neff, A.W., King, M.W. & Mescher, A.L. Dedifferentiation and the role of sall4 in reprogramming and patterning during amphibian limb regeneration. *Dev. Dyn.* **240,** 979–989 (2011).

40) Knapp, D. *et al.* Comparative transcriptional profiling of the axolotl limb identifies a tripartite regeneration-specific gene program. *PLoS One* **8,** e61352 (2013).

41) Stewart, R. *et al*. Comparative RNA-seq analysis in the unsequenced axolotl: the oncogene burst highlights early gene expression in the blastema. *PLoS Comput. Biol.* **9,** e1002936 (2013).

42) Monaghan, J. R. *et al.* 2012. Gene expression patterns specific to the regenerating limb of the Mexican axolotl. *Biol. Open.* **1,** 937–948 (2012).

43) Carlson, M.R., Bryant, S.V. & Gardiner, D.M. Expression of *Msx-2* during development, regeneration, and wound healing in axolotl limbs. *J. Exp. Zool.* **282,** 715–723(1998).

44) Koshiba, K. *et al.* Expression of Msx genes in regenerating and developing limbs of axolotl. *J. Exp. Zool.* **282,** 703-714 (1998).

45) Kumar, A., Velloso, C.P., Imokawa, Y. & Brockes, J.P. The regenerative plasticity of isolated urodele myofibers and its dependence on Msx1. *PLoS Biol.* **2,** e218 (2004).

46) Satoh, A., Bryant, S.V. & Gardiner, D.M. Regulation of dermal fibroblast dedifferentiation and redifferentiation during wound healing and limb regeneration in the axolotl. *Dev. Growth Differ.* **50,** 743-754 (2008).

47) McEwan, J., Lynch, J. & Beck, C. Expression of key retinoic acid modulating genes suggests active regulation during development and regeneration of the amphibian limb. *Dev. Dyn.* **240,** 1259-1270 (2011).

48) Kochegarov, A., Moses-Arms, A., Hanna, M.C. & Lemanski, L.F. Identification of genes involved in limb regeneration in the axolotl *Ambystoma mexicanum*. *JSM Regen. Med.* **3,** 1014 (2015).

49) McCusker, C.D. *et al.* Positional plasticity in regenerating *Amybstoma mexicanum* limbs is associated with cell proliferation and pathways of cellular differentiation. *BMC Dev. Biol.* **15,**

45 (2015).

50) Imokawa, Y. & Yoshizato, K. Expression of sonic hedgehog gene in regenerating newt limbs. *Wound Repair Regen.* **6,** 366–370 (1998).

51) Torok, M.A., Gardiner, D.M., Izpisua-Belmonte, J.C. & Bryant, S.V. Sonic hedgehog (shh) expression in developing and regenerating axolotl limbs. *J. Exp. Zool.* **284,** 197-206 (1999).

52) Nacu, E. *et al.* FGF8 and SHH substitute for anterior-posterior tissue interactions to induce limb regeneration. *Nature* **533,** 407-410 (2016).

53) Satoh, A. *et al.* Analysis of scleraxis and dermo-1 genes in regenerating limb of *Xenopus laevis*. *Dev. Dyn.* **235**, 1065-73 (2006).

54) Christensen, R.N., Weinstein, M. & Tassava, R.A. Fibroblast growth factors in regenerating limbs of Ambystoma: cloning and semi-quantitative RT-PCR expression studies. *J. Exp. Zool.* **290,** 529–540 (2001).

55) Han, M.-J., An, J.-Y. & Kim, W.-S. Expression patterns of Fgf-8 during development and limb regeneration of the axolotl. *Dev. Dyn.* **220,** 40–48 (2001).

56) Christensen, R.N., Weinstein, M. & Tassava, R.A. Expression of fibroblast growth factors 4, 8, and 10 in limbs, flanks, and blastemas of Ambystoma. *Dev. Dyn.* **223,** 193-203 (2002).

57) Knapp, D. *et al.* Comparative transcriptional profiling of the axolotl limb identifies a tripartite regeneration-specific gene program. *PLoS One* **8,** e61352 (2013).

58) Girich, A.S., Isaeva, M.P. & Dolmatov, I.Y. Wnt and frizzled expression during regeneration of internal organs in the holothurian Eupentacta fraudatrix. *Wound Repair Regen.* **25,** 828-835 (2017).

Supplementary Table S2. Sequences corresponding to *Newtic1* in other vertebrates (Blast results with E: <e-5)

Species	Genome database[59]				
	Genome	cDNAs	cDNAs *ab initio*	Peptide	Peptide *ab initio*
Human (*Homo sapiens*)	ND	ND	ND	ND	ND
Opossum (*Monodelphis domestica*)	ND	ND	ND	ND	ND
Chicken (*Gallus gallus*)	ND	ND	ND	ND	ND
Anole lizard (*Anolis carolinensis*)	ND	ND	ND	ND	ND
Frog (*Xenopus tropicalis*)	ND	ND	ND	ND	ND
Coelacanth (*Latimeria chalumnae*)	ND	ND	ND	ND	ND
Zebrafish (*Danio rerio*)	ND	ND	ND	ND	ND
	Transcriptome database				
Axolotl (*Ambystoma mexicanum*)[10,60]	Hit (ID: AMEXTC_0340000020965)				
Red spotted newt (*Notophthalmus viridescens*)[61,62]	Hit (ID: com1037174_c0_seq1)				
Iberian ribbed newt (*Pleurodeles waltl*)[11,63)]	Hit (Reconstructed in this study)				

References

59) ENSEMBL genome database; http://asia.ensembl.org/info/data/ftp/index.html
60) Axolotl-Omics.org; https://www.axolotl-omics.org/
61) http://sandberg.cmb.ki.se/redspottednewt/
62) Abdullayeva, I. *et al*. A reference transcriptome and inferred proteome for the salamander Notophthalmus viridescens. *Exp. Cell Res.* **319,** 1187–1197 (2013).
63) Supplementary Figure S5.

Supplementary Table S3 (provided as a separate Excel file)**.** Mapping results showing expression levels (normalized FPMK) of secretory molecules in whole blood.

Supplementary Table S4. PCR primer sets and amplicon size.

gene	Primer Sequences		Amplicon Size (bp)	Cycle number
	Forward	Reverse		
RARb	TCCCAAAGTCCGAGGTCAAG	AGAAGGAGCCGATAAAACAGGA	181	40
LHX2	TTCAAACACCACCAGTTGCG	CTCCTGTGTTTTCTTGGCGT	188	40
WNT 9A	GCATTCCACATAGCAGCACC	GGACTACAGACCTGACCTAT	200	40
SALL 1	TTGCTTGTCAGAGTGCCTTG	TGCTGCTTCAAATTACCCTTT	112	40
ZFHX4	GGCTTTCCCAAATACCTCAGC	GGAACATCTTTGGACACACAGG	144	40
MSX1	TCTCTACATGGCTTCTGTCCA	CTTGTCCGGCTCCTCGTC	112	40
TWIST2	ATCATCCCCACACTGCCCT	GCTTGAGGGTCTGGATCTTAC	52	40
CRABP1	TGCACACAACATCATCAGC	GAGATCAACTTCAAAATCGGGGA	209	40
SHH	GGCTTCGACTGGGTCTACTT	AATCCTCACCCCTTGCTCC	135	40
SCXB	GCACCAACAGCGTCAACA	GGTGGGAGATGTAGCTGGAG	181	40
FGF10	ATGTTTCTTCTTGTGAGGCGT	CACCCTTCCTCAGTACCCAT	112	40
FRZD1	GCCATGCCGAAGAAGTAGA	AGCGGCCCATCATCTTCCT	190	40
EF1a	GACCTTTGCCCCCAGTAACGTAACCAC	ACTGGGTGTTGCTGGCGCTACTTCTTG	573	25
NEWTIC1 - Fig. 1c	CTTGTGAGGATTACATTGTGCA	GCTCGTGTGAAACCTAAGAGA	1,512	35
NEWTIC1 - Fig. 1c	GGTTGGTCGAAAAGAGTCAGA	GCCCTATGCATGAAAGTTGG	76	30
NEWTIC1 - Fig. 1g	TGGTGCTGGGATGGAAGTTGAATTT	GCTCTGATGTCCACGGCCTCTAT	795	38
NEWTIC1 - Fig. 2c; Fig. 6b	TTTCCCACCATGCACCTCCTCGTA	CCTTCCTGTGGGCTCTGATGTCCA	497	35
TGFβ1	GTAGTTGTGGACAGACATTGGGCGTGT	TTGTAAAGCACCATGACTTCCTCGGGC	433	30
BMP2	AGAATAACAAGGCACCAGAACACGCCA	ATCCCGGCCACCATTGTCAACCTAAAG	511	30
VEGFC	GTGATTATGCCCCATTCCTGCTCTGCT	CACACCGGTTCCTTCTGTTGTATCCCC	650	30
PDGFC	CGATGAGTATATCCCCTCCGCTCCTGG	GCCGTCTAGCCTTCTGTATTCCCTTTGC	606	30
IGF II	GTTCATTTCCGCCTGTCCGATGTCTCT	GCTCTGAAGGTGCCCAAGATGACTGTG	584	30
nsCCN	GACTGCAATGACCTACGACCCTGTGAC	TCTTCCCACCCTTGATGACCGTGTACT	1,096; 658	25
COL-a	GAATGAGAGAGACCGAGAGAGTGACCA	CAGAAAAGCTCCGTGAAATGCAGCAAT	505	35
COL-b	AAGCTGATGGAGTGGTAGGCCCTGTAG	TTTGGATTGGAGGTCACTGGGAAGCTG	628	35
MMP21	GGTAATCGCCCAGTTCTCCATTCGCTT	ATCAACCAGAACTCACCCTCAGTCCCA	957	35
MMP9	CTCACCTTCACTCAGATCTACAGCGGC	TTGGGTCTTCACGTCCAACCTCCAGTA	1,389	30

Supplementary Table S5. Antibodies for immunolabelling.

Antibody	Dilution	Provider
**Primary**		
Rabbit anti-Newtic1 polyclonal antibody	1:200	Custom made; Merck (Sigma-Aldrich), Tokyo, Japan
Rabbit anti-RFP polyclonal antibody	1:500	600-401-379; Rockland Immunochemicals, PA 19468, USA
Rabbit anti-TGFb1 polyclonal antibody	1:500	CYT4632; Celltechgen LLC, TX 77079, USA
Rabbit anti-BMP2 polyclonal antibody	1:500	LS-B13128; LifeSpan BioSciences, InC., WA 98121, USA
Mouse anti-vimentin monoclonal antibody	1:500	ab28028; Abcam,Tokyo, Japan
**Secondary**		
Alexa Fluor 488-conjugated goat anti-mouse IgG (H + L) polyclonal antibody	1:500	A11001; Thermo Fisher Scientific, Tokyo, Japan
Rhodamine (TRITC)-conjugated affiniPure goat anti-rabbit polyconal antibody	1:500	111-025-003; Jackson ImmunoResearch Laboratories, PA 19390, USA
Biotinylated goat anti-rabbit IgG polyclonal antibody	1:500	BA-1000; Vector laboratories, CA 94010, USA

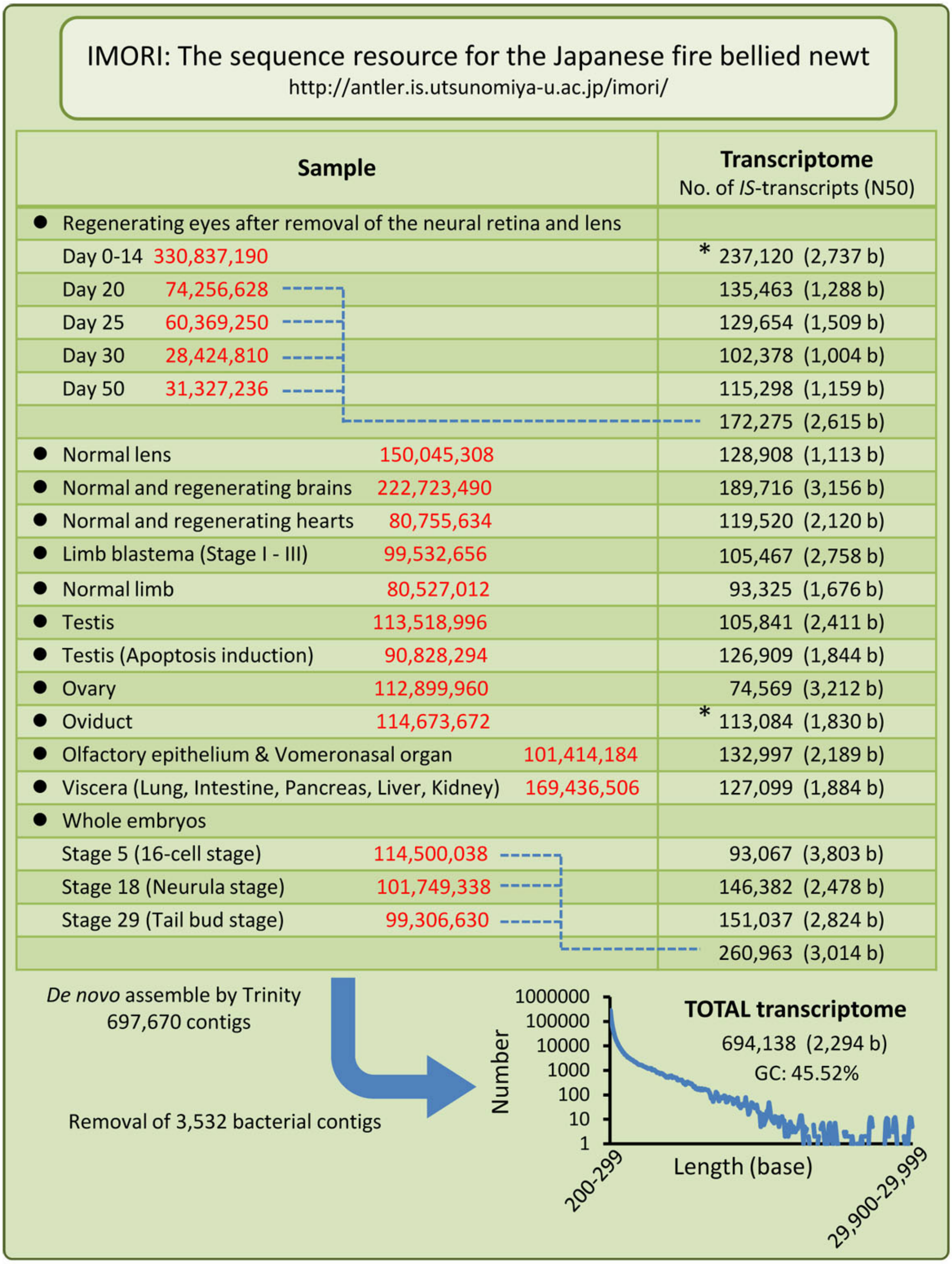

Supplementary Figure S1. TOTAL transcriptome in IMORI. For the study of the Japanese fire-bellied newt *Cynops pyrrhogaster*, the Japan newt research community (JNRC) opened the sequence resource site ‘IMORI’ to the public in 2014 (http://antler.is.utsunomiya-u.ac.jp/imori/)[26]. In contrast to other newt species, *C. pyrrhogaster*

has been studied across a broad spectrum of life science fields such as gametogenesis[64], reproduction[65-67], fertilization[68,69], embryogenesis[70,71], metamorphosis[72], regeneration[3,5-7,25-27,29,73], chemical sense and behavior[74-77], toxicology[78], evolution[79,80], and ecology and conservation[81,82]. Therefore, IMORI is diligently updated to provide *de novo* transcriptome databases in response to demands from each research area (21 databases at present). In this study, we constructed a comprehensive transcriptome database named 'TOTAL'. We assembled, using Trinity (see Methods), all of the clean reads (2,177,126,832 x 101 bases) which had been collected to construct 21 databases in IMORI (NCBI: SRP034152), removed bacterial sequences from the resulting 697,670 contigs through annotation by blastx (E-value <e-5) with the Nr database in NCBI (namely Nr annotation), and finally obtained 694,138 contigs (i.e., *in silico* transcripts (*IS*-transcripts)). The length distribution of these *IS*-transcripts ranged up to 29,971 bases with an N50 value of 2,294 bases. 169,309 *IS*-transcripts (36,742 non-redundant transcripts) in TOTAL were Nr annotated (E-value <e-5), and 63,216 *IS*-transcripts (19,367 non-redundant transcripts) of them were further mapped with Gene Ontology (GO) terms by blast2GO (E-value <e-6) and classified into functional categories. IMORI provides these results (xlsx) as well as the list of *IS*-transcripts in TOTAL (fasta). Asterisks: Database which has been validated for its availability in other studies[26,65]

References

64) Eto, K. *et al.* Reduced expression of an RNA-binding protein by prolactin leads to translational silencing of programmed cell death protein 4 and apoptosis in newt spermatogonia. *J. Biol. Chem.* **284,** 23260-23271 (2009).

65) Yokoe, M. *et al.* Sperm proteases that may be involved in the initiation of sperm motility in the newt, *Cynops pyrrhogaster*. *Int. J. Mol. Sci.* **15,** 15210-15224 (2014).

66) Yokoe, M. *et al.* A novel cysteine knot protein for enhancing sperm motility that might facilitate the evolution of internal fertilization in amphibians. *PLoS One* **11,** e0160445 (2016).

67) Kon, S. *et al.* Sperm storage influences the potential for spontaneous acrosome reaction of the sperm in the newt *Cynops pyrrhogaster*. *Mol. Reprod. Dev.* **84,** 1314-1322 (2017).

68) Ueno, T. *et al.* Egg activation in physiologically polyspermic newt eggs: involvement of IP $_3$ receptor, PLCγ, and microtubules in calcium wave induction. *Int. J. Dev. Biol.* **58,** 315-323 (2014).

69) Mutua, J. *et al.* Functional diversity of voltage-sensing phosphatases in two urodele amphibians. *Physiol. Rep.* **2**, e12061 (2014).

70) Takano, K. *et al.* Development of Ca^{2+} signaling mechanisms and cell motility in presumptive ectodermal cells during amphibian gastrulation. *Dev. Growth Differ.* **53,** 37-47 (2011).

71) Yanagi, T. *et al.* The Spemann organizer meets the anterior-most neuroectoderm at the equator of early gastrulae in amphibian species. *Dev. Growth Differ.* **57,** 218-231 (2015).

72) Chiba, C. *et al.* Metamorphosis inhibition: an alternative rearing protocol for the newt *Cynops pyrrhogaster*. *Zool. Sci.* **29,** 293-298 (2012).

73) Sousounis, K. *et al.* A robust transcriptional program in newts undergoing multiple events of lens regeneration throughout their lifespan. *eLife* **4,** e09594 (2015).

74) Inoue, R. & Nakatani K. Changes in olfactory response to amino acids in Japanese newts after transfer from aquatic to terrestrial habitat. *Zool. Sci.* **27,** 369-373 (2010).

75) Iwata, T. *et al.* Responsiveness of vomeronasal cells to a newt peptide pheromone, sodefrin as monitored by changes of intracellular calcium concentrations. *Peptides* **45,** 15-21 (2013).

76) Nakada, T. *et al.* Expression of G proteins in the olfactory receptor neurons of the newt

Cynops pyrrhogaster: their unique projection into the olfactory bulbs. *J. Comp. Neurol.* **522,** 3501-3519 (2014).

77) Nakada, T. *et al.* Imorin: A sexual attractiveness pheromone in female red-bellied newts (*Cynops pyrrhogaster*) *Sci. Rep.* **7,** 41334 (2017).

78) Kudo, Y. *et al.* Dietary administration of tetrodotoxin and its putative biosynthetic intermediates to the captive-reared non-toxic Japanese fire-bellied newt, *Cynops pyrrhogaster*. *Toxicon* **137**, 78-82 (2017).

79) Tominaga, A. *et al.* Phylogeny and historical demography of *Cynops pyrrhogaster* (Amphibia: Urodela): taxonomic relationships and distributional changes associated with climatic oscillations. *Mol. Phylogenet. Evol.* **66,** 654-667 (2013).

80) Tominaga, A. *et al.* Genomic displacement and shift of the hybrid zone in the Japanese fire-bellied newt. *J. Hered.* **109,** 232-242 (2018).

81) Tominaga, A., Matsui, M. & Kokuryo, Y. Occurrence and evolutionary history of two *Cynops pyrrhogaster* lineages on the Izu Peninsula. *Curr. Herpetol.* **34,** 19-27 (2015).

82) Tominaga, A. *et al.* Origin and genetic uniformity of introduced population of *Cynops pyrrhogaster* (Amphibia: Urodela) on Hachijojima Island. *Curr. Herpetol.* **35,** 64-68 (2016).

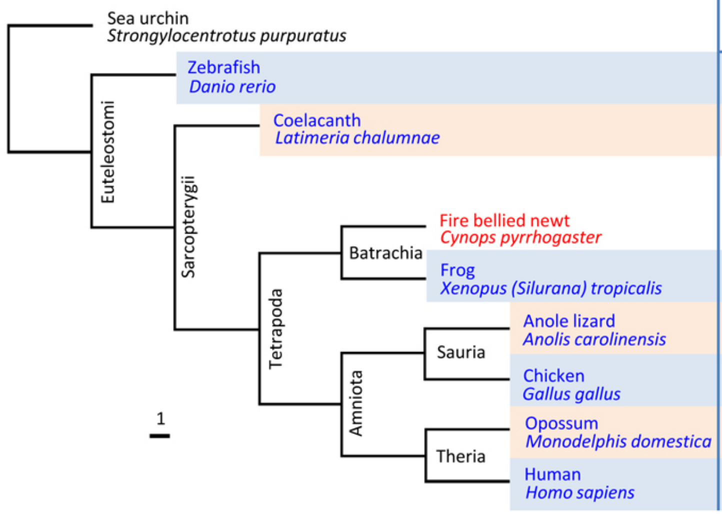

Species	Total # of peptides (# of genes)	tblastn: % peptides corresponding to *Cp* transcripts (% genes)		blastx: % transcripts in *Cp* TOTAL, corresponding to other vertebrates' peptides	
		E < e-5	E < e-10	E < e-5	E < e-10
Zebrafish *Danio rerio*	43,761 (24,674)	92.9 (94.6)	90.7 (92.8)	19.5	15.5
Coelacanth *Latimeria chalumnae*	23,434 (14,507)	98.2 (99.4)	97.1 (98.8)	21.6	16.9
Frog *Xenopus (Silurana) tropicalis*	23,539 (15,227)	98.7 (99.3)	98.0 (98.9)	17.7	14.1
Anole lizard *Anolis carolinensis*	19,110 (15,498)	96.8 (98.8)	95.6 (98.1)	20.5	9.7
Chicken *Gallus gallus*	17,691 (13,722)	94.0 (97.3)	92.7 (96.2)	11.0	10.6
Opossum *Monodelphis domestica*	22,244 (16,475)	94.3 (98.1)	92.4 (96.8)	15.3	12.5
Human *Homo sapiens*	70,076 (20,635)	84.6 (91.8)	79.4 (89.7)	14.1	12.0

Supplementary Figure S2. Comparisons between TOTAL and reference proteomes of other vertebrates (UniProt; http://www.uniprot.org/proteomes/) by tblastn and blastx. TOTAL contained *IS*-transcripts (or mRNA sequence information) corresponding to a large proportion of protein-coding genes in other vertebrates, for example, ~99% in coelacanth, ~99% in frog (*Xenopus tropicalis*), ~96% in chicken and ~90% in human, indicating that almost all of the protein-coding genes are common (or homologous) between the newt and other tetrapods. Thus, TOTAL seemed likely to work as a comprehensive reference transcriptome database for the newt. On the other hand, more than 75% of *IS*-transcripts in TOTAL did not have homology (E-value ≥e-5) to nucleotides/proteins of other organisms. These sequences would contain mRNA of unique genes (our target in this study) as well as an untranslated region (UTR) of mRNA and non-coding RNA.

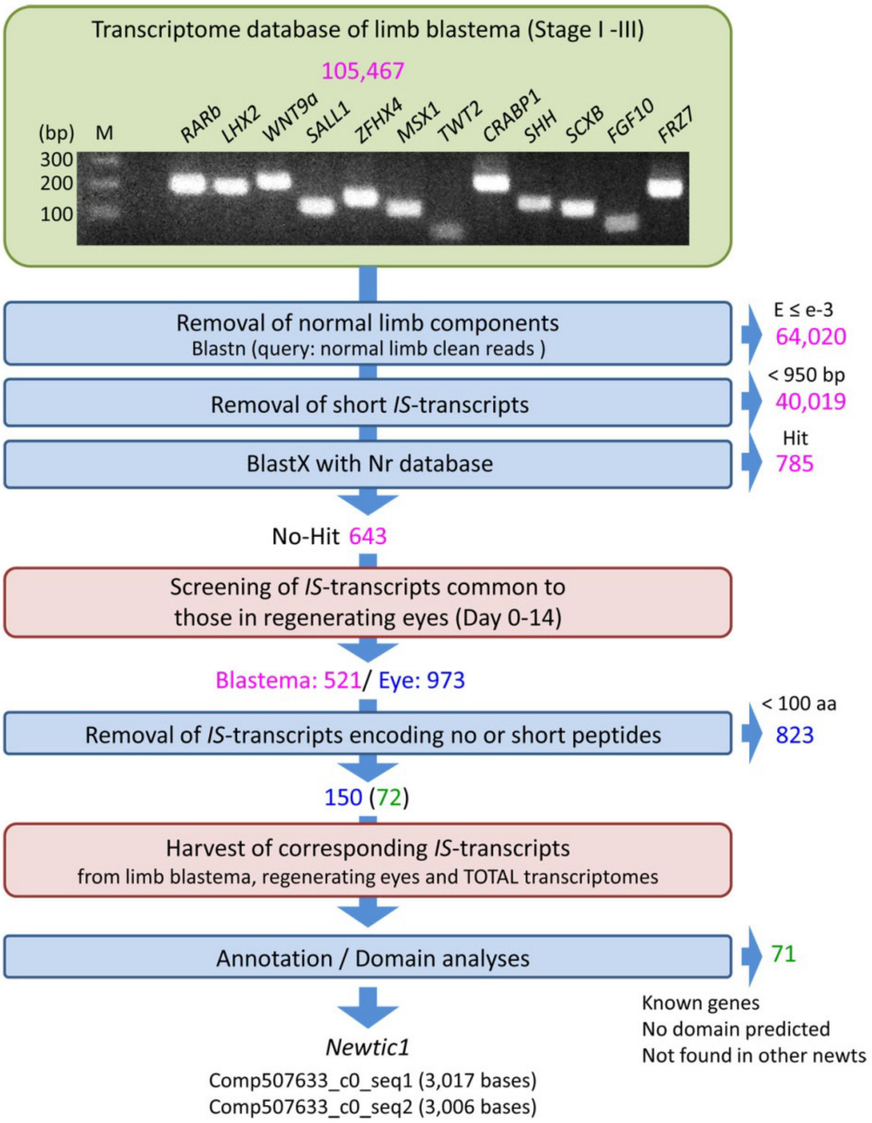

Supplementary Figure S3. Work flow showing the screening of unique genes whose quantity of transcripts increased in the adult newt limb blastema. We started from the transcriptome (105,464 *IS*-transcripts) of the limb blastema at stages I to III in IMORI. This transcriptome contained sequence information of genes that have been suggested to participate in limb regeneration (see the inset PCR results; Supplementary Table S1). We screened *IS*-transcripts (≥ 950 bases) whose expression might have been up-regulated in the blastema by subtracting normal limb components from the blastema transcriptome. We then filtered Nr-annotated *IS*-transcripts out of the resulting 1,428 *IS*-transcripts. From the remaining 643 No-Hit *IS*-transcripts, we screened 72 candidate protein-coding genes whose *IS*-transcripts (150 in total) also existed in the transcriptome of regenerating eyes (Day 0-14). We harvested *IS*-transcripts corresponding to 72 candidate genes from the limb blastema, regenerating eyes and TOTAL transcriptomes, manually screened two unique candidate genes through Nr annotation and domain analysis (InterProScan sequence search; https://www.ebi.ac.uk/interpro/search/sequence-search), and finally obtained one that also existed in other newt species (Supplementary Table S2). Two *IS*-transcripts of *Newtic1* were found in TOTAL (Supplementary Figure S4).

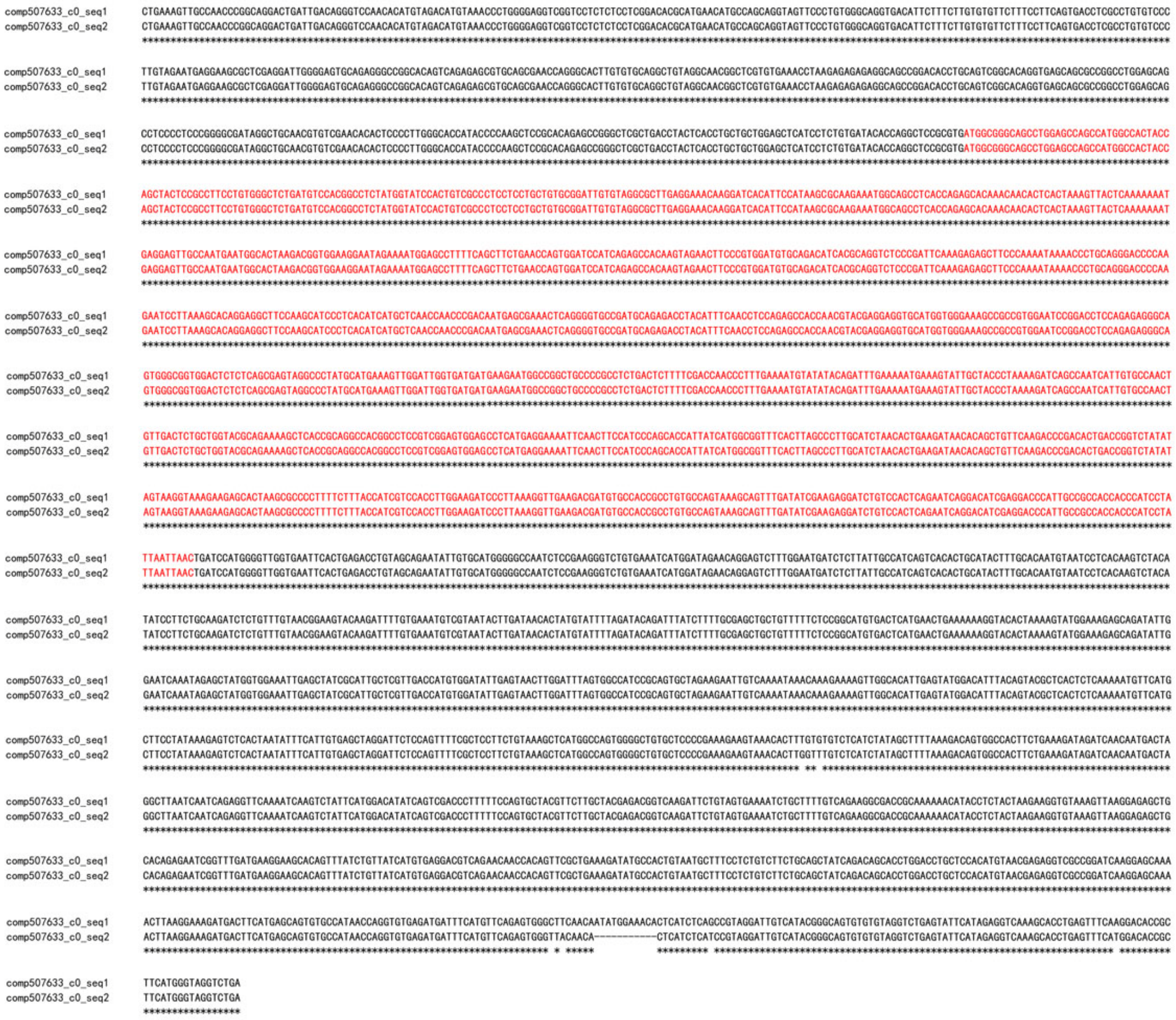

Supplementary Figure S4. Alignment of two *Newtic1* contigs in the TOTAL transcriptome database. The sequences shown here are a reverse complement of the original. Red shows the coding sequence.

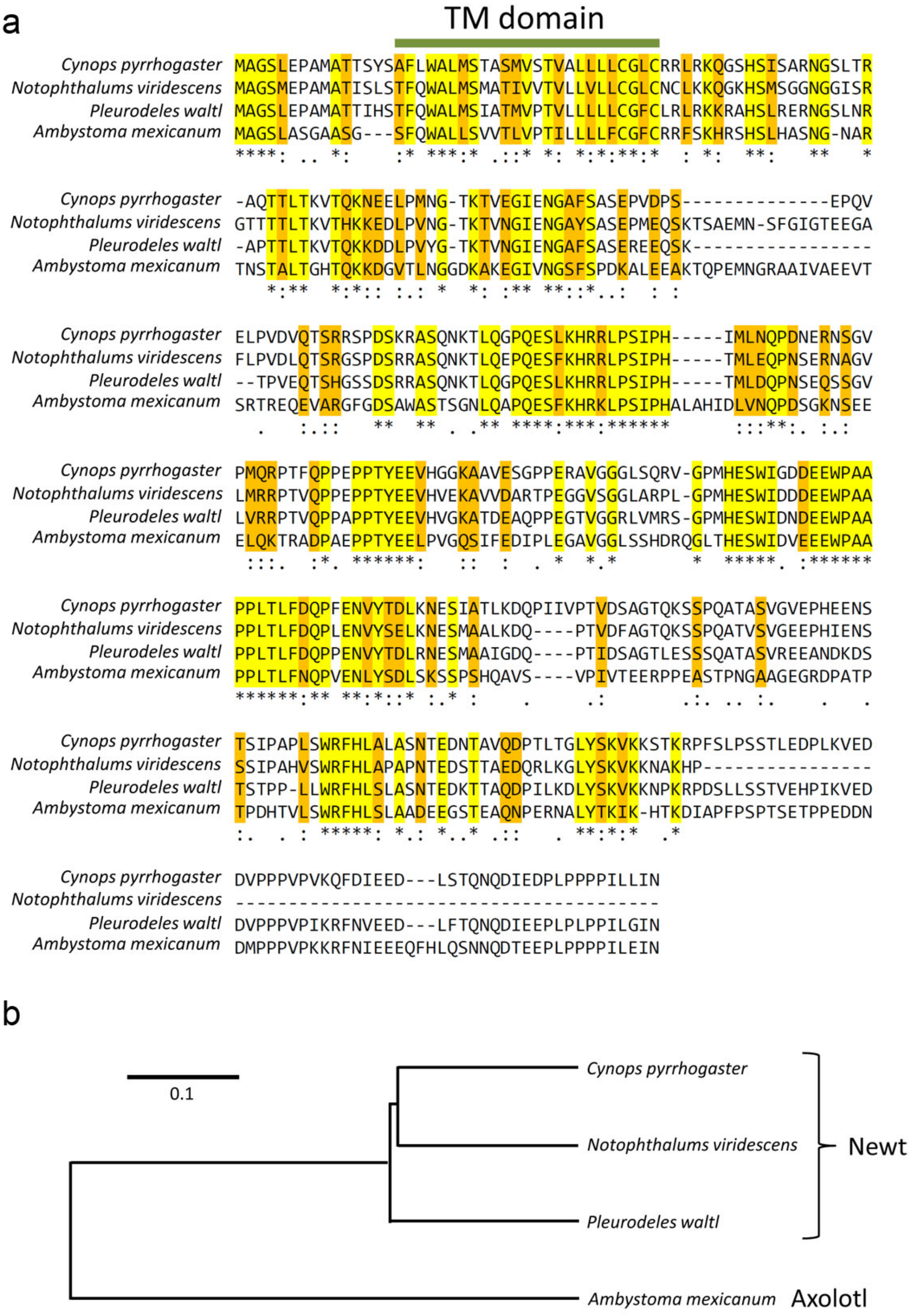

Supplementary Figure S5. Comparison of Newtic1 protein among urodele species. **(a)** Multiple alignment by ClastalW. TM: transmembrane domain predicted by InterProScan (https://www.ebi.ac.uk/interpro/search/sequence-search). **(b)** Phylogenetic relationships deduced by neighbor-joining method. For contigs of *Notophthalmus viridescens*, *Pleurodeles waltl* and *Ambystoma mexicanum*, see Supplementary Table S2.

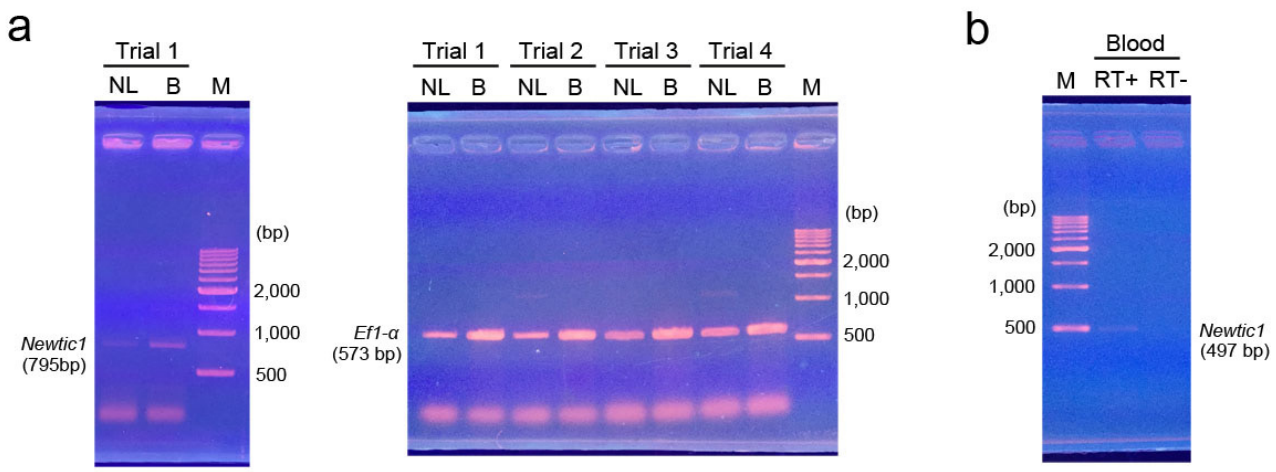

Supplementary Figure S6. Full-length gels from which the data in main figures were obtained. **(a)** Gels of the data in Fig. 1g. The lanes of Trial 1 were used. **(b)** Gel of the data in Fig. 2c.

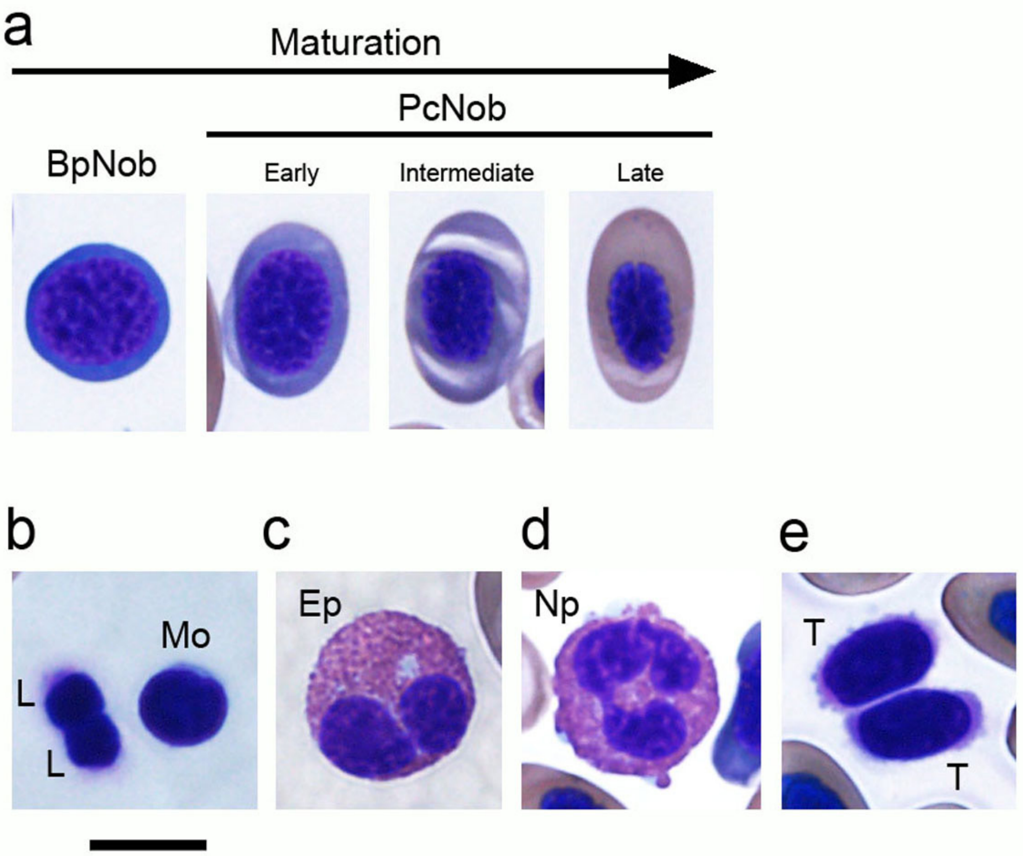

Supplementary Figure S7. Wright-Giemsa stain of blood smear in the newt *C. pyrrhogaster*. Peripheral blood was obtained from adult animals by amputation of their forearms. **(a)** Nucleated erythrocytes (normoblasts) at different developmental stages. The proportion of normoblasts in all blood cells was 83-94% (n=9). Normoblasts were divided into basophilic normoblasts (BpNobs) and polychromatic normoblasts (PcNobs). BpNobs are an immature state of normoblasts, which are characterized by their oblate spheroid shape with a big round polychromatic nucleus and a narrow cytoplasmic space. The cytoplasm is stained in blue. PcNobs are defined as normoblasts in a transitional to mature state. The proportion of BpNobs and PcNobs in all normoblasts was less than 2.2% (1.1 ± 0.3%, n=9) and 97.8-100% (98.9 ± 0.3%, n=9), respectively. During the development of PcNobs, they change shape, becoming flattened ellipsoid (oval/oblong) and their nucleus becomes more compact. Concomitantly, their cytoplasmic space gradually increases. The cytoplasmic stain also changed from grey to pink/orange as they matured while synthesizing hemoglobin. In this study, we subdivided PcNobs into early, intermediate and late stages: PcNobs at intermediate and late stages, which looked similar in morphology, were grey and pink/orange in color with the cytoplasmic stain, respectively; PcNobs at an early stage had a larger and rounder nucleus, and had less cytoplasmic space that was stained blue. However, in experiments in which we carried out immunocytochemistry using blood cell suspensions, we divided PcNobs into two groups (early and intermediate/late) because it was difficult to differentiate between PcNobs at intermediate and late stages according to their morphological characteristics only. The proportion of early PcNobs and intermediate/late PcNobs in all PcNobs was 22.1 ± 2.2% (n=9) and 77.9 ± 2.2% (n=9), respectively. In particular experimental conditions in which almost all PcNobs became slightly

swollen and transparent, we occasionally recognized a small number of PcNobs at a late stage whose shape and color were preserved, and that never showed Newtic1 immunoreactivity (Fig. 2b). These cells are presumably in a mature state, corresponding to orthochromatic normoblasts (OcNobs). We estimated the incorporation of such presumptive OcNobs into intermediate/late PcNobs as less than 3.8% (n=3). Consequently, the ratio of normoblasts in the adult newt was roughly BpNobs : early PcNobs : intermediate/late PcNobs (OcNobs) = 1 : 22 : 77 (<3.8). **(b)** Monocyte (Mo) and lymphocytes (L). **(c)** Eosinophil (Ep). **(d)** Neutrophil (Np). **(e)** Thrombocytes (T). Scale bar, 20 µm.

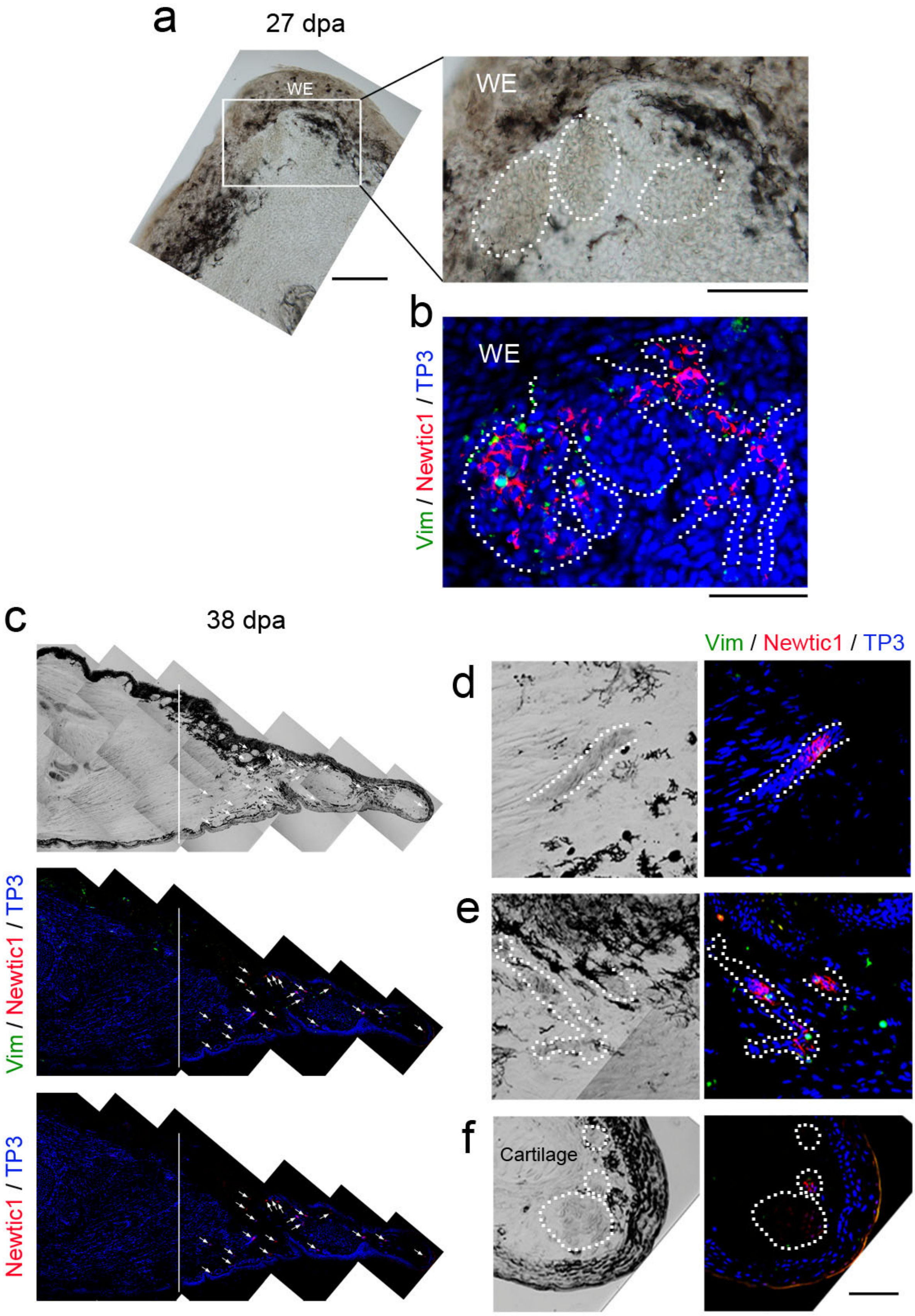
a
27 dpa
WE
WE
b
WE
Vim / Newtic1 / TP3
c
38 dpa
Vim / Newtic1 / TP3
Newtic1 / TP3
Vim / Newtic1 / TP3
d
e
f
Cartilage

Supplementary Figure S8. EryCs are transported through regenerating capillaries/vessels. **(a)** A section showing thick blood vessels (dotted lines) in the protrusion of the blastema at 27 days post amputation (dpa). WE: wound epidermis. Scale bars, 300 µm (left) and 200 µm (right). **(b)** A section showing the presence of EryCs in regenerating capillaries/vessels (dotted lines) in the protrusion of the blastema at 27 dpa. Regenerating capillaries/vessels formed the loops (or network) with large ventricles, in which EryCs accumulated to form larger Newtic1+ aggregates. Scale bar, 100 µm. **(c)** Representative image showing the distribution pattern of EryCs (arrows) in a regenerating forearm at 38 dpa (n=3). Vertical line: amputation plane. EryCs were mostly distributed within the regenerate, although the size and density of Newtic1+ aggregates decreased compared to those in the blastema at stage III (Fig. 4a). Immunoreactivity of the region proximal to the amputation plane recovered as in an intact limb. Scale bar, 1 mm. **(d-f)** Enlargement of EryCs in **c**. EryCs seemed to be flowing along blood capillaries/vessels (dotted lines). Note that EryCs at this stage were always accompanied by monocytes as observed in normal blood (Fig. 3). Scale bar, 100 µm.

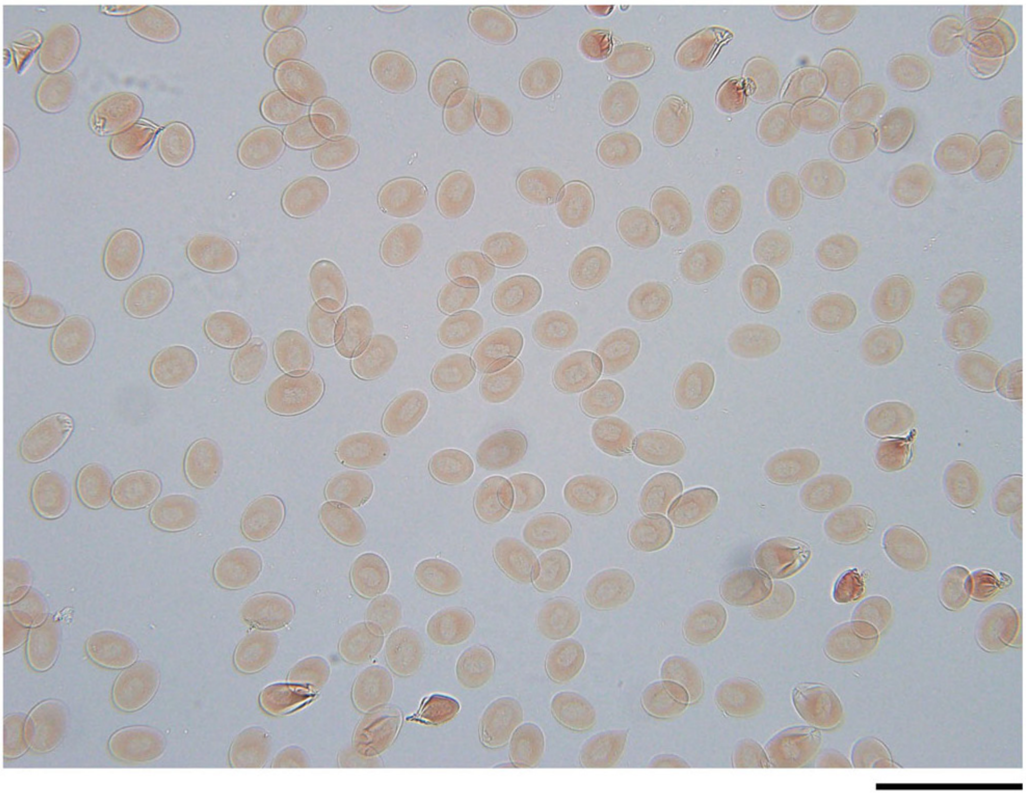

Supplementary Figure S9. Representative image showing PcNobs purified from normal blood by the procedure described in Fig. 6b. Scale bar, 100 µm.

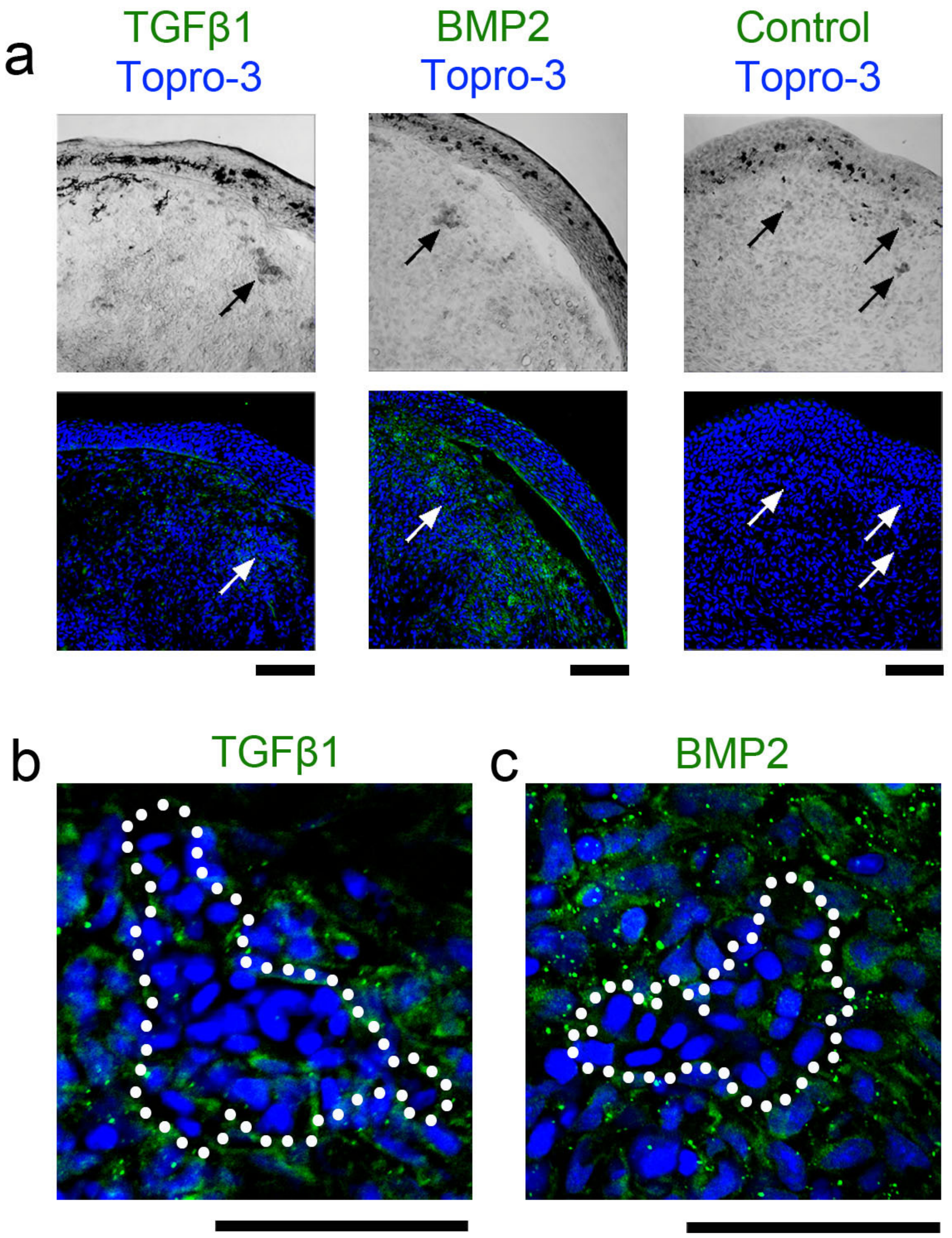

Supplementary Figure S10. Representative images showing TGFβ1- and BMP2-immunoreactivities in EryCs in a growing blastema (n=3). To examine whether fixation conditions (Zamboni's fixative, 4°C, 6 hrs) applied to Fig. 8 may be responsible for decreased immunoreactivities in both growth factors in EryCs, we changed the conditions to 4% paraformaldehyde at 22°C for 2 hrs, which was applied to blood cell suspensions in Fig. 7. We used regenerating limbs in a transitional stage from stage II to stage III. **(a)** Immunoreactivities in the protrusion of a blastema. Arrows point to EryCs. Topro-3: nucleus. Control: RFP. Scale bars, 200 µm. **(b)** TGFβ1 immunoreactivity in EryCs (arrow) in **a**. **(c)** BMP2 immunoreactivity in EryCs (arrow) in **a**. Dotted line: wall of the blood vessel. As a result, distribution patterns of labeling were substantially the same as those in Fig. 8, although signal intensity decreased. Scale bars, 100 µm.

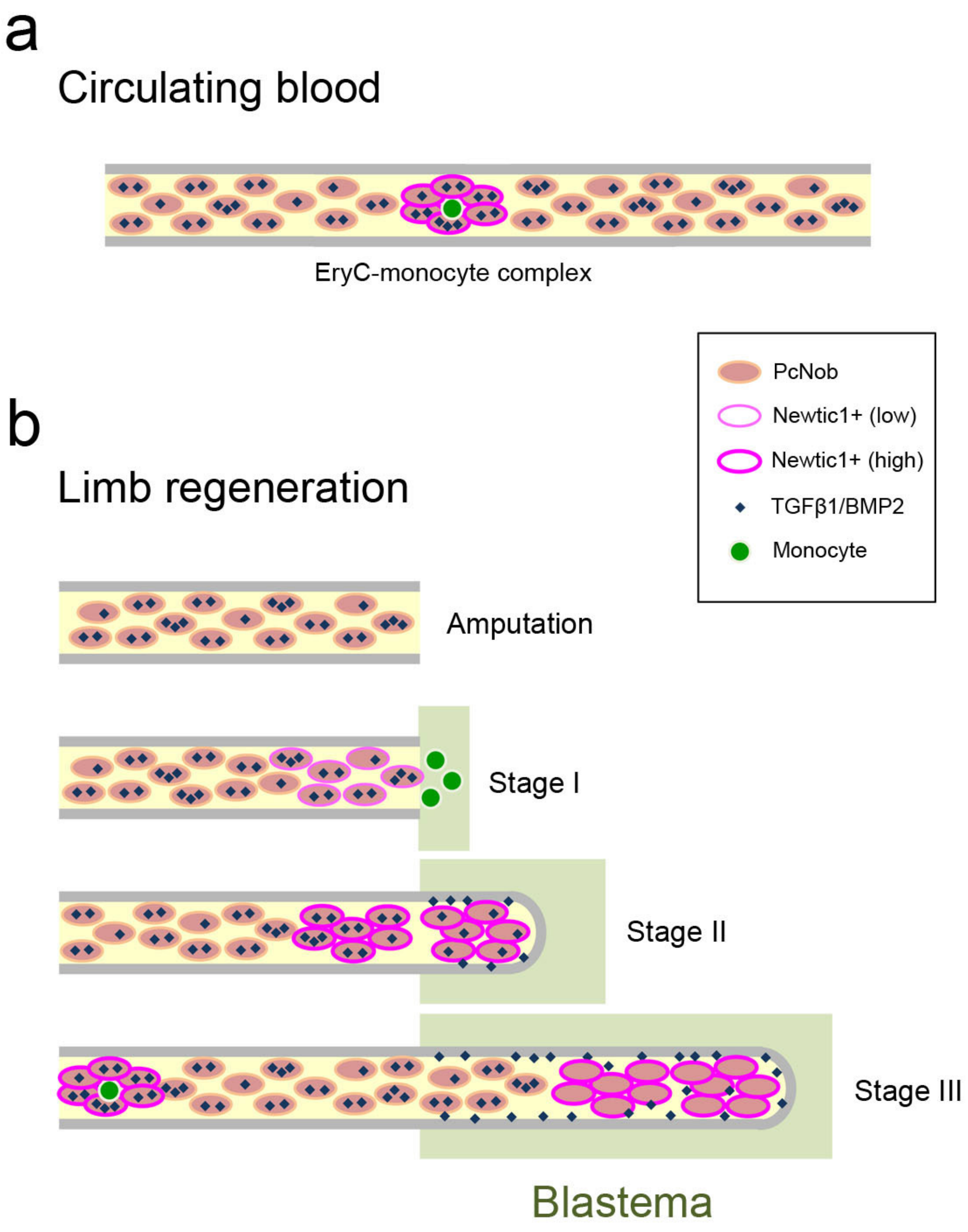

Supplementary Figure S11. Summary. **(a)** Circulating blood. Newtic1+ PcNobs form EryC-monocyte complexes, and circulate together with free Newtic1- PcNobs. PcNobs and EryCs carry secretory molecules such as TGFβ1 and BMP2. **(b)** Limb regeneration. After the wound closes, PcNobs near the amputation site start to express Newtic1 (stage I). Newtic1+ PcNobs form EryCs. EryCs are translocated into the growing blastema as the capillaries/vessels regenerate (stage II). EryCs accumulate in the distal part of the growing blastema as angiogenesis proceeds (stage III). PcNobs in EryCs seem to release TGFβ1 and BMP2, both of which are then attached to the surface (along the equatorial plane) of PcNobs or matrices along the endothelium of capillaries/vessels.
